# Heterogeneity of immune checkpoint inhibitor-related inflammatory central nervous system adverse event reporting signals in primary and metastatic brain tumors: a pharmacovigilance study with single-cell and spatial transcriptomic contextualization

**DOI:** 10.3389/fimmu.2026.1866830

**Published:** 2026-07-08

**Authors:** Junlin Song, Zeyu He, Chong Han, Xiaohong Hou

**Affiliations:** Department of Neurosurgery, Affiliated Hospital of Zunyi Medical University, Zunyi, Guizhou, China

**Keywords:** central nervous system immune-related adverse events, immune checkpoint inhibitors, pharmacovigilance, single-cell RNA sequencing, spatial transcriptomics

## Abstract

**Background:**

Immune checkpoint inhibitors (ICIs) can induce immune-related adverse events (irAEs) across multiple organ systems. Although inflammatory central nervous system irAEs (CNS inflammatory irAEs) are uncommon, they are often severe. Primary CNS tumors and brain metastases have distinct immune microenvironments, yet the heterogeneity of ICI-related inflammatory CNS irAE reporting signals across tumor phenotypes remains poorly understood.

**Methods:**

We used pharmacovigilance signal discovery, external corroboration, and transcriptomic contextualization of pre-existing brain tumor immune landscapes. We constructed an ICI-exposed cohort from the FDA Adverse Event Reporting System (FAERS) and compared inflammatory CNS irAE disproportionality signals across primary CNS tumors, brain metastases, and non-CNS solid tumors. External comparison used the Japanese Adverse Drug Event Report database (JADER). Public single-cell RNA sequencing datasets were analyzed to characterize baseline strict inflammatory and broad stress-related modules across cellular compartments, with spatial transcriptomics used as secondary descriptive visualization in brain metastasis tissue.

**Results:**

In FAERS, inflammatory CNS irAE reporting signals suggested tumor phenotype-associated heterogeneity, with adjusted odds ratios of 1.65 (95% CI, 1.02–2.65) for primary CNS tumors and 3.12 (95% CI, 2.45–3.98) for brain metastases versus non-CNS solid tumors. Signals were stronger under a strict noninfectious phenotype and attenuated under a broad neuroinflammatory phenotype. Thyroid comparator analyses showed no comparable enrichment, whereas the myocarditis-related comparator was too sparse in the brain metastasis subgroup for meaningful inference. JADER showed a broadly similar pattern, although primary CNS tumor estimates were sparse and exploratory. Baseline single-cell analyses localized strict inflammatory module activity mainly to myeloid and T/NK compartments, while spatial maps served only as secondary descriptive visualization.

**Conclusions:**

ICI-related inflammatory CNS irAE reporting signals suggested tumor phenotype-associated differences, most prominently in brain metastases. Stricter phenotype definitions appeared more specific than broader neuroinflammatory definitions. Public single-cell datasets characterized pre-existing immune-rich myeloid/T-NK compartments, while spatial maps provided only secondary descriptive tissue-level visualization and did not demonstrate irAE-onset tissue states. These pharmacovigilance findings should be interpreted as hypothesis-generating reporting associations, not evidence of incidence, absolute risk, or causality.

## 1 Introduction

Immune checkpoint inhibitors (ICIs) have profoundly reshaped the therapeutic landscape across multiple malignancies (1, 2). However, by augmenting antitumor immune responses, they may also trigger immune-related adverse events (irAEs) involving multiple organ systems (3). Neurologic irAEs are relatively uncommon overall, yet they are often clinically severe and pose major challenges in differential diagnosis and clinical management (4). In particular, inflammatory events involving the central nervous system (CNS), such as encephalitis, meningitis, and demyelinating syndromes, may result in disabling or even fatal outcomes in a subset of patients (5). As the indications for ICIs continue to expand, earlier recognition and more accurate characterization of ICI-related CNS toxicity have become pressing priorities in the safety evaluation of cancer immunotherapy (6).

Compared with pan-cancer populations, patients with primary CNS tumors and brain metastases (BrM) have highly distinctive immunologic backgrounds. On the one hand, primary brain tumors, exemplified by glioblastoma (GBM), typically display a profoundly immunosuppressive tumor microenvironment (TME), with myeloid enrichment and limited T-cell infiltration (7, 8). On the other hand, brain metastasis is not simply a process of tumor cell seeding in the brain parenchyma; rather, it is accompanied by dynamic remodeling of resident brain immune cells, such as microglia, and infiltrating peripheral immune populations, particularly tumor-associated macrophages and T-cell subsets (9). Single-cell and spatial studies have revealed heterogeneous immune niches in primary and metastatic brain tumors, suggesting that brain tumor backgrounds should not be treated as immunologically uniform contexts (10).

However, existing studies of ICI-related neurologic irAEs have largely described phenotypic spectra, severity, and clinical management from a pan-cancer perspective or in the form of clinical reviews (11). Systematic, multidimensional evidence remains limited regarding whether inflammatory CNS irAEs display phenotype-specific reporting signal enrichment in primary CNS tumors and brain metastases, two disease settings with markedly different immune microenvironments, and whether different phenotype definitions can better distinguish more specific inflammatory reporting phenotypes from nonspecific tissue stress or injury-related signals (12). This issue is particularly important in patients with brain tumors, in whom infection, cerebral edema, radiation-related injury, metabolic disturbances, and tumor progression itself may all contribute to neurologic symptoms and imaging abnormalities, thereby blurring the distinction between broad neurotoxicity and more stringently defined immune-inflammatory phenotypes (13).

Against this background, we combined FAERS-based pharmacovigilance signal discovery, external comparison in JADER (14), and transcriptomic contextualization of baseline brain tumor immune landscapes. We evaluated inflammatory CNS irAE reporting signals across primary CNS tumors, brain metastases, and non-CNS solid tumors; then used public single-cell datasets to characterize compartment-level strict inflammatory and broad stress module patterns. Spatial transcriptomic data from brain metastases were used only as secondary descriptive visualization of these single-cell-derived module patterns. These transcriptomic analyses were intended to contextualize, not validate or mechanistically explain, the pharmacovigilance findings.

## 2 Materials and methods

### 2.1 Study design and data sources

This study combined pharmacovigilance signal discovery, external database comparison, and transcriptomic contextualization. FAERS was used as the primary discovery database, JADER as an independent comparison database, and public single-cell and spatial transcriptomic datasets as exploratory contextual resources for baseline brain tumor immune landscapes. The transcriptomic analyses were not intended to validate causality or reproduce tissue states during irAE onset; the spatial analysis was considered supportive and descriptive rather than inferential.

### 2.2 Data sources

The primary FAERS analysis included reports from January 2014 through December 2025 and integrated the DEMO, DRUG, REAC, INDI, OUTC, THER, and RPSR core tables. The JADER corroboration analysis followed the same general logic and used an independent national pharmacovigilance database for directional external corroboration. For reports with multiple versions of the same case, only the latest version was retained. These covariates were selected because they were consistently available and clinically relevant across FAERS and JADER. Additional clinical variables, including disease stage, performance status, prior radiotherapy, corticosteroid exposure, and detailed concomitant medications, were not consistently structured or reliably available across the spontaneous reporting databases and therefore could not be incorporated into the primary multivariable models.

ICI exposure was identified using a prespecified drug dictionary covering cytotoxic T-lymphocyte-associated protein 4 (CTLA-4), programmed cell death protein 1 (PD-1), and programmed death-ligand 1 (PD-L1) inhibitors, together with their common brand names and generic name synonyms. In the primary analysis, exposure was defined by agents coded as primary suspect (PS); secondary suspect (SS) drugs were included only in supplementary sensitivity analyses. Tumor background was classified by pattern matching within the indication text and categorized as primary CNS tumor, brain metastasis, or non-CNS solid tumor.

Two prespecified outcome phenotypes were used. The narrow, high-specificity inflammatory CNS irAE phenotype primarily included predefined Preferred Terms (PTs) for encephalitis, aseptic meningitis, meningoencephalitis, myelitis, and transverse myelitis. The broad neuroinflammatory phenotype additionally included encephalopathy, brain edema, demyelination, and other broader CNS inflammation/injury-related terms. A strict noninfectious narrow phenotype and a negative-control thyroid irAE phenotype were also defined to assess specificity and robustness. Because thyroid irAEs are usually detected through routine laboratory testing and may not adequately capture surveillance or diagnostic-workup bias relevant to neurologic events, we additionally defined a myocarditis-related non-CNS serious irAE comparator phenotype. This comparator was intended to probe whether brain tumor-related cohorts showed nonspecific enrichment for serious irAEs requiring clinical suspicion and diagnostic evaluation, rather than enrichment specific to inflammatory CNS phenotypes. The myocarditis-related comparator included MedDRA Preferred Terms related to myocarditis, immune-mediated myocarditis, autoimmune myocarditis, myopericarditis, and related myocarditis terms. This analysis was interpreted as a diagnostic-workup comparator rather than as a definitive negative control.

Pharmacovigilance analyses used a case-noncase design. The primary disproportionality metric was the reporting odds ratio (ROR) with 95% confidence intervals, supplemented by the information component (IC). Multivariable logistic regression models were constructed using non-CNS solid tumors as the reference group to assess adjusted reporting associations of primary CNS tumors and brain metastases with the target phenotypes. To reduce overfitting given sparse outcome counts, particularly in the primary CNS tumor subgroup, the multivariable models were restricted to prespecified covariates with sufficient completeness and clinical relevance. When sparse outcomes or low expected frequencies were present, Firth penalized logistic regression was preferentially used to reduce small-sample bias. Sensitivity analyses included the strict noninfectious narrow phenotype, the broad phenotype, restriction to healthcare professional (HCP) reports, stratification by ICI treatment regimen, and time-to-onset analyses. JADER analyses used the same tumor background classification, outcome definitions, and regression framework as FAERS to assess qualitative cross-database agreement.

### 2.3 Single-cell RNA sequencing analysis

To characterize pre-existing immune niche features that could contextualize the pharmacovigilance signals, we analyzed the public single-cell RNA sequencing datasets GSE131928 (15) and GSE131907 (16). These datasets were not derived from patients with clinically adjudicated CNS irAEs and were therefore used to describe baseline tumor immune architecture rather than irAE-specific tissue states. In this context, “baseline” refers to publicly available tumor transcriptomic datasets not sampled at the time of CNS irAE onset, rather than necessarily pre-ICI treatment specimens. GSE131928 contains both Smart-seq2 and 10x Chromium data from isocitrate dehydrogenase (IDH)-wild-type GBM and was analyzed as two independent evidence streams representing high-coverage and high-throughput platforms, respectively. Brain metastasis (mBrain) samples from GSE131907 were extracted to provide a comparative microenvironmental dataset for brain metastases. Given the intrinsic differences between Smart-seq2 and 10x platforms in capture efficiency, detection rate, and quantification, the two platforms were processed separately rather than jointly integrated, and cross-platform consistency was assessed at the level of overall patterns.

Raw single-cell matrices were processed in Python 3 using pandas, numpy, scanpy, anndata, scipy, statsmodels, matplotlib, and seaborn. AnnData objects were first constructed, after which mitochondrial genes were identified and the total counts, number of detected genes, and mitochondrial transcript fraction were calculated for each cell. Low-quality cells were excluded based on dataset-specific hard thresholds for quality-control metrics. For the Smart-seq2 dataset, cells with detected gene numbers outside the range of 3, 000 to 8, 800 were removed. For the 10x Genomics dataset, cells were retained if they contained between 300 and 4, 950 detected genes and had a mitochondrial transcript fraction below 7.5%. These platform-specific thresholds were inspected to remove low-quality outliers without excluding the main cellular distribution. Expression matrices were then normalized and log-transformed. Highly variable genes were selected using dataset-specific variance ranking, with the top 3000 genes retained for downstream dimensionality reduction. Principal component analysis was performed using the top 30 principal components, nearest-neighbor graphs were constructed using 15 neighbors, and Uniform Manifold Approximation and Projection (UMAP) embeddings were generated. Unsupervised clustering was performed using the Leiden algorithm with a resolution parameter of 0.5. For the large GSE131907 matrix, chunk-wise reading and sparse matrix reconstruction were used to reduce memory burden.

Cell-type annotation was based on clustering results, canonical markers reported in the literature, and the original dataset annotations. To improve cross-platform and cross-dataset comparability, major populations were harmonized into the following primary analytical categories: Myeloid, T/NK, Astrocyte-like, OPC/Oligodendrocyte-like, Malignant-like, and Vascular-like. Non-core compartments, such as B cells, mast cells, and fibroblasts, were retained in extended annotations but were not included in the principal comparative analyses.

At the transcriptional module level, we predefined two compact, literature-informed but non-irAE-specific gene modules: a strict inflammatory module and a broad stress module. The strict inflammatory module was constructed to represent a relatively focused immune-inflammatory program, including IFN/interferon-stimulated signaling, antigen processing and presentation, inflammatory chemotaxis, and inflammatory activation or adhesion. The broad stress module was constructed to represent broader nonspecific stress or injury-related programs, including hypoxia, angiogenic/metabolic stress, immediate early stress-response transcription, and heat-shock, unfolded-protein, or injury-associated stress responses. The composition of these modules was guided by canonical gene-set and pathway concepts, including knowledge-based gene-set analysis, MSigDB Hallmark immune-inflammatory and stress-related programs, IFN-γ/STAT1-driven immune activation, inflammatory chemokine programs, hypoxia-associated transcriptional responses, and cellular adaptive stress-response programs. The primary strict inflammatory module included CXCL9, CXCL10, CXCL11, CCL2, CCL5, STAT1, IRF1, IFIT1, IFIT3, B2M, TAP1, CD74, HLA-DRA, HLA-DRB1, TNF, TNFAIP3, NFKBIA, ICAM1, VCAM1, and IL1B. The primary broad stress module included HIF1A, VEGFA, CA9, LDHA, SLC2A1, ADM, SERPINE1, JUN, FOS, ATF3, DDIT3, HSPB1, HSPA1A, CXCL8, and PTGS2. Alternative module definitions used in sensitivity analyses are provided in **Supplementary Table S7**. These modules were not intended to serve as irAE-specific diagnostic signatures, but rather as predefined transcriptional contrasts for comparing relatively focused immune-inflammatory activity with broader nonspecific stress/injury-related activity in public non-irAE brain tumor datasets (17–22).

Module scores were computed using scanpy.tl.score_genes to generate strict_inflam_score and broad_stress_score, and these were then standardized within each dataset to yield z-scores. A composite exploratory metric, strict-minus-broad, was defined as strict_inflam_z − broad_stress_z. This subtraction-based metric was used to describe the relative predominance of the strict inflammatory module over the broad stress module on a common within-dataset standardized scale. We chose subtraction rather than a ratio because z-standardized module scores can include zero and negative values, making ratio-based measures unstable and difficult to interpret. The strict-minus-broad metric was therefore interpreted only as a relative descriptive contrast, not as an independent mechanistic biomarker. Differences in module scores across cell types were first evaluated globally using the Kruskal-Wallis test, followed by pairwise Mann-Whitney U tests with Benjamini-Hochberg correction for multiple comparisons. These analyses were intended primarily to describe distributional differences in module scores across cellular compartments.

To assess whether the observed compartment-level patterns depended on the specific primary gene lists or on the subtraction-based strict-minus-broad metric, we performed sensitivity analyses across the GSE131928 Smart-seq2, GSE131928 10x, and GSE131907 mBrain single-cell datasets using alternative literature-informed inflammatory and stress-related gene modules. We also calculated residual-based relative inflammatory scores by regressing the corresponding inflammatory z-score on the corresponding stress z-score within each dataset and using the residuals as alternative measures of inflammation-dominant activity after accounting for broad stress activity. The sensitivity results were summarized at the compartment level and compared with the primary strict-minus-broad metric.

### 2.4 Spatial transcriptomic analysis

Spatial transcriptomic analyses were performed using the public Visium brain metastasis dataset GSE179572 (23). Raw GEO files were first organized into a standard Visium directory structure, including filtered_feature_bc_matrix.h5 and the corresponding spatial folder containing tissue images, coordinate files, and scale-factor metadata. Samples were then read individually using scanpy.read_visium, and sample-specific prefixes were added to barcodes to prevent naming collisions.

Spatial data processing was also performed in Python 3 using pandas, numpy, scanpy, anndata, scipy, matplotlib, and seaborn. For each sample, mitochondrial genes were identified and the total counts, number of detected genes, and mitochondrial transcript fraction were calculated for each spot. Only in_tissue = 1 spots were retained. Adaptive sample-specific filtering thresholds based on MAD were then applied: spots were excluded if total counts or detected gene numbers were below the sample-specific median minus 3 MADs, or if mitochondrial transcript fraction exceeded the sample-specific median plus 3 MADs. Low-information genes expressed in only a very small number of spots were then removed, followed by total-count normalization and log1p transformation.

The same primary strict inflammatory and primary broad stress modules used in the single-cell analyses and listed in **Supplementary Table S7** were applied in the spatial transcriptomic analyses. For each spot, strict_inflam_score and broad_stress_score were calculated and standardized as z-scores, and the strict-minus-broad metric was derived accordingly. To facilitate spatial compartment interpretation, myeloid-like, T/NK-like, tumor-like, vascular-like, and oligo-like compartment-like signatures were additionally defined and scored continuously using gene program scores. Because each Visium spot reflects a local mixture of signals rather than a pure single-cell population, the main figures in the manuscript emphasize continuous spatial score maps rather than forcing each spot into a single cell-type assignment. For interpretive support, simplified niche annotations were generated according to the relative dominance of immune-rich, tumor-rich, vascular-like, and oligo-like signatures. Spots with a maximum standardized signature score below 0.20 were labeled as Low-signal. Spots were labeled as Mixed if the difference between the highest and second-highest standardized signature scores was below 0.35. All remaining spots were assigned to the dominant niche category. These thresholds were used only for simplified visualization and were not used for formal spatial inference. These annotations were used only to aid interpretation of the high-scoring regions in the continuous module maps and were not treated as the primary inferential framework. Representative samples were used to visualize the spatial juxtaposition of the strict inflammatory module, broad stress module, strict-minus-broad composite metric, and immune- or tumor-related signatures, whereas the continuous maps and simplified niche maps for all samples were provided as **Supplementary Material**. No formal spatial-neighborhood, ligand-receptor, or distance-based statistical inference was performed; therefore, the spatial analysis was interpreted as descriptive visualization of tissue-level module organization rather than as an independent mechanistic or inferential layer.

### 2.5 Statistical analysis and software

Pharmacovigilance analyses were performed primarily in R, whereas single-cell and spatial transcriptomic analyses were performed primarily in Python 3. Continuous variables are presented as medians and interquartile ranges, and categorical variables as counts and percentages. Baseline categorical characteristics across tumor phenotype groups were compared using Pearson’s chi-square test. P-values were not calculated for cohort totals or complementary category rows. Unless otherwise specified, all statistical tests were two-sided, with P < 0.05 considered statistically significant. For multiple pairwise comparisons at the single-cell level, the Benjamini-Hochberg method was used to control the false discovery rate. Because spontaneous reporting databases do not provide true exposure denominators or standardized follow-up information, results from FAERS and JADER were interpreted as reporting associations and hypothesis-generating evidence rather than estimates of true incidence or strict causal effects. Accordingly, terms such as “signal, “ “association, “ “heterogeneity, “ and “phenotype-associated pattern” refer to disproportionality in spontaneous reporting rather than clinical incidence, absolute risk, or mechanistic causality. The single-cell and spatial transcriptomic analyses were used primarily to characterize baseline immune and stress-related microenvironmental patterns in publicly available primary and metastatic brain tumor datasets. These analyses were considered exploratory and contextual; they were not intended to establish causality, validate pharmacovigilance signals, or directly model the tissue state during CNS irAE onset.

## 3 Results

### 3.1 FAERS cohort construction and baseline characteristics

Within the ICI-exposed cohort derived from FAERS, 2, 145 reports involved primary CNS tumors, 5, 630 involved brain metastases, and 85, 210 involved non-CNS solid tumors, yielding a final analytical cohort of 92, 985 reports (**Table 1**; **Supplementary Table S1**). Within the primary CNS tumor cohort, GBM accounted for 67.6% of reports and was the dominant diagnostic subtype; the remainder included other glioma-lineage tumors and a small number of other primary brain tumors (**Table 2**).

**Table 1:** Baseline characteristics of ICI-exposed reports by tumor phenotype.

Characteristics	Primary CNS tumor	Brain metastases	Non-CNS solid tumor	P-value
Total Cohort, N	2, 145	5, 630	85, 210	-
Age >= 65, n (%)	1, 200 (55.9)	3, 150 (55.9)	42, 100 (49.4)	0.012
Sex: Male, n (%)	1, 340 (62.5)	3, 400 (60.4)	52, 100 (61.1)	0.145
Reporter: HCP, n (%)	1, 850 (86.2)	4, 900 (87.0)	68, 100 (79.9)	0.001
Regimen: Anti-PD-1 Monotherapy	1, 900 (88.6)	4, 500 (79.9)	70, 000 (82.2)	<0.001
Regimen: Combination Therapy	245 (11.4)	1, 130 (20.1)	15, 210 (17.8)	-

HCP, Healthcare Professional; ICI, Immune Checkpoint Inhibitor. P-values were calculated using Pearson’s chi-square test. P-values were not calculated for total cohort size or for combination therapy because these rows represent. cohort totals or complementary regimen categories.

**Table 2:** Composition of the primary CNS tumor cohort by diagnostic subtype.

Diagnostic subtype	Number of reports (n)	Percentage (%)
Glioblastoma (GBM)	1, 450	67.6
Other Glioma Lineage	510	23.8
Other Primary CNS Tumors	185	8.6
Total Primary CNS Cohort	2, 145	100.0

Notes: Defined using prespecified indication-term lexicons mapped to diagnostic subgroup categories.

The three tumor background groups differed in several baseline characteristics. The proportion of patients aged >=65 years was 55.9% in both the primary CNS tumor and brain metastasis cohorts, compared with 49.4% in the non-CNS solid tumor cohort. Reports submitted by healthcare professionals (HCPs) accounted for 86.2% and 87.0% of reports in the primary CNS tumor and brain metastasis cohorts, respectively, versus 79.9% in the non-CNS solid tumor cohort. In terms of treatment regimen, anti-PD-1 monotherapy predominated in primary CNS tumors, whereas the proportion receiving combination therapy was higher in brain metastases (**Table 1**).

### 3.2 Overall inflammatory CNS irAE reporting signals suggest tumor phenotype-associated heterogeneity

**Figure 1:**
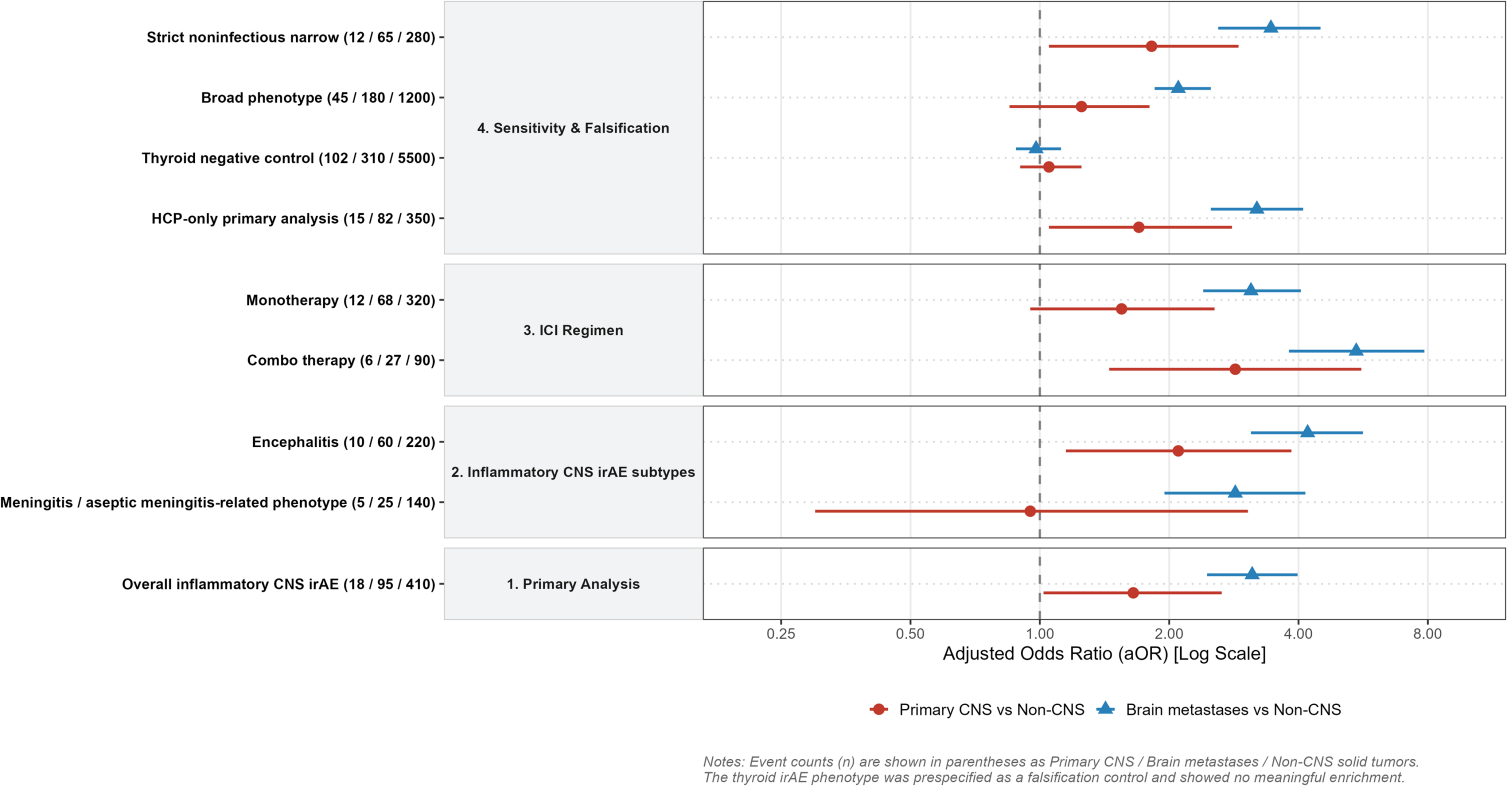
Phenotype-specific adjusted odds ratios for inflammatory CNS irAE reporting signals after ICI exposure across tumor phenotypes.

In the primary narrow inflammatory CNS irAE analysis, both primary CNS tumors and brain metastases had elevated disproportionality signals relative to non-CNS solid tumors, although the magnitude of reporting disproportionality differed between groups. The primary CNS tumor cohort included 18 target events, with an ROR of 1.75 (95% CI, 1.08–2.82) and an IC of 0.78 (95% CI, 0.05–1.51). The brain metastasis cohort included 95 target events, with an ROR of 3.55 (95% CI, 2.85–4.42) and an IC of 1.80 (95% CI, 1.45–2.15), suggesting a more prominent disproportionality signal in the setting of brain metastases. By comparison, the non-CNS solid tumor reference group included 410 target events (**Table 3**; **Figure 1**).

**Table 3:** Overall disproportionality signals for inflammatory CNS irAEs across tumor phenotypes.

Phenotype	Total reports (N)	CNS irAE cases (n)	ROR (95% CI)	IC (95% CI)
Primary CNS Tumor	2, 145	18	1.75 (1.08-2.82)	0.78 (0.05-1.51)
Brain Metastases	5, 630	95	3.55 (2.85-4.42)	1.80 (1.45-2.15)
Non-CNS Solid Tumor (Reference)	85, 210	410	1.00 (Ref)	-

ROR, Reporting Odds Ratio; IC, Information Component. Significance is defined as lower 95% CI > 1 (ROR) and > 0 (IC).

Multivariable models yielded a similar overall reporting pattern. Using non-CNS solid tumors as the reference group, the adjusted odds ratio (aOR) was 1.65 (95% CI, 1.02–2.65) for primary CNS tumors and 3.12 (95% CI, 2.45–3.98) for brain metastases, suggesting that the adjusted reporting associations between tumor phenotype and inflammatory CNS irAE reports remained after accounting for age, sex, reporting year, reporter type, and treatment regimen (**Table 4**). In the full model, combination therapy, HCP reporting, and later reporting year were associated with increased event reporting, whereas age and sex were not significantly associated (**Supplementary Table S2**). Overall, reporting signals were more prominent in brain metastases, whereas the primary CNS tumor estimate was based on only 18 events and should be interpreted as hypothesis-generating.

**Table 4:** Phenotype-specificity, falsification, and robustness analyses.

Analysis strategy/phenotype	Primary CNS cases (n)	Brain Mets cases (n)	Non-CNS cases (n)	Primary CNS aOR (95% CI) vs non-CNS	Brain Mets aOR (95% CI) vs non-CNS	Consistency interpretation
1. Primary narrow inflammatory CNS irAE	18	95	410	1.65 (1.02-2.65)	3.12 (2.45-3.98)	Moderate reporting signal
2. Strict noninfectious narrow	12	65	280	1.82 (1.05-2.90)	3.45 (2.60-4.50)	Reporting signal more prominent
3. Broad neuroinflammatory	45	180	1200	1.25 (0.85-1.80)	2.10 (1.85-2.50)	Reporting signal attenuated
4. Negative-control thyroid irAE	102	310	5500	1.05 (0.90-1.25)	0.98 (0.88-1.12)	No comparable reporting enrichment
5. Myocarditis-related non-CNS serious irAE comparator	3	1	1847	0.33 (0.11–1.03)	0.34 (0.05–2.43)	Underpowered and statistically uninformative for brain metastases because of a single event; interpreted descriptively only
6. HCP-only primary analysis	15	82	350	1.70 (1.05-2.80)	3.20 (2.50-4.10)	Directionally maintained

Notes: aOR, adjusted odds ratio derived from multivariable logistic regression adjusted for age, sex, reporting year, reporter type, and treatment regimen. The negative-control phenotype did not show a comparable generalized reporting enrichment pattern. The myocarditis-related comparator was used as a non-CNS serious irAE phenotype requiring clinical suspicion and diagnostic work-up. Because the brain metastasis myocarditis comparator included only one event, the corresponding estimate was underpowered and statistically uninformative; this analysis was interpreted descriptively and was not used as evidence against CNS-specific surveillance bias.

### 3.3 Reporting signals are more prominent under stricter inflammatory phenotypes and attenuated under broad phenotypes

In prespecified robustness analyses, the strict noninfectious narrow phenotype yielded more prominent adjusted reporting associations than the primary narrow phenotype. In primary CNS tumors, the aOR increased from 1.65 to 1.82 (95% CI, 1.05–2.90), whereas in brain metastases it increased from 3.12 to 3.45 (95% CI, 2.60–4.50). By contrast, when the outcome definition was broadened to the broad neuroinflammatory phenotype, the association weakened to 1.25 (95% CI, 0.85–1.80) in primary CNS tumors and to 2.10 (95% CI, 1.85–2.50) in brain metastases, suggesting attenuation of the reporting signal (**Table 4**; **Figure 1**).

Thyroid irAEs showed no comparable reporting enrichment in either primary CNS tumors or brain metastases. HCP-restricted analyses preserved the direction of the primary analysis. The myocarditis-related non-CNS serious irAE comparator was also examined as a diagnostic-workup comparator: events occurred in 3 of 867 primary CNS tumor reports, 1 of 211 brain metastasis reports, and 1, 847 of 109, 546 non-CNS solid tumor reports, with adjusted ORs of 0.33 (95% CI, 0.11–1.03) and 0.34 (95% CI, 0.05–2.43), respectively (**Table 4**). Because the brain metastasis estimate was based on a single myocarditis event, this comparator analysis was severely underpowered and statistically uninformative for the brain metastasis subgroup; it was therefore interpreted descriptively and not used as evidence against surveillance bias. Additional sensitivity analyses are summarized in **Supplementary Table S3**.

Taken together, these findings suggest phenotype-specific inflammatory CNS reporting enrichment rather than generalized irAE reporting enrichment, although the myocarditis comparator could not meaningfully assess surveillance bias in the brain metastasis subgroup because of the single event count. However, because neither thyroid irAEs nor myocarditis-related comparator events directly capture CNS-specific surveillance intensity, these analyses cannot exclude the possibility that more intensive neurologic monitoring among patients with brain metastases contributed to the higher inflammatory CNS reporting signals.

### 3.4 Subtype, regimen, onset, and seriousness analyses

Subtype-stratified analyses are provided in **Supplementary Tables S4**, **S5**. In primary CNS tumors, subtype counts were very small, including 10 encephalitis, 5 meningitis, and 3 myelitis-related events; therefore, these estimates were considered descriptive and underpowered rather than analytically weighted main findings. Subtype-level estimates were more informative in brain metastases, where event counts were larger.

Regimen-stratified analyses suggested more prominent inflammatory CNS irAE reporting signals under combination therapy. Under monotherapy, the RORs were 1.55 (95% CI, 0.95–2.55) for primary CNS tumors and 3.10 (95% CI, 2.40–4.05) for brain metastases. Under PD-1/PD-L1 plus CTLA-4 combination therapy, these increased to 2.85 (95% CI, 1.45–5.60) and 5.45 (95% CI, 3.80–7.85), respectively (**Table 5**; **Figure 1**).

**Table 5:** Effect modification by ICI regimen across tumor phenotypes.

Therapy regimen	Primary CNS: n, ROR (95% CI)	Brain Mets: n, ROR (95% CI)	Non-CNS (Ref): n
Anti-PD-1/PD-L1 Monotherapy	12, 1.55 (0.95-2.55)	68, 3.10 (2.40-4.05)	320
Anti-PD-1 + Anti-CTLA-4 Combo	6, 2.85 (1.45-5.60)	27, 5.45 (3.80-7.85)	90

Combo refers to Anti-PD-1/PD-L1 plus Anti-CTLA-4 therapy.

Time-to-onset analyses showed that inflammatory CNS irAEs were reported with shorter time-to-onset intervals in primary CNS tumors and brain metastases than in non-CNS solid tumors. Median time to onset was 45 days (IQR, 21–95) for primary CNS tumors and 38 days (IQR, 18–80) for brain metastases, compared with 65 days (IQR, 30–120) for non-CNS solid tumors. Weibull shape parameters also suggested a greater tendency toward earlier onset in the former two groups (β= 0.85 and 0.92), whereas non-CNS solid tumors showed an increasing hazard trend (β= 1.15) (**Table 6**). Seriousness analyses further showed that inflammatory CNS irAE reports in brain tumor settings more frequently included serious outcomes. Compared with non-CNS solid tumors, the proportions of death, life-threatening events, and hospitalization were all higher in primary CNS tumors and brain metastases. For example, death occurred in 16.7%, 17.9%, and 8.5% of cases, respectively, and hospitalization in 77.8%, 82.1%, and 65.1%, respectively. When further stratified by subtype, encephalitis- and myelitis-related events showed relatively high rates of death or life-threatening outcomes in both primary CNS tumors and brain metastases, suggesting that, within spontaneous reports, these event subtypes were more frequently accompanied by serious outcomes (**Table 7**; **Supplementary Table S6**).

**Table 6:** Time-to-onset and Weibull pattern analyses of inflammatory CNS irAEs.

Phenotype	Median TTO, days (IQR)	Weibull shape parameter (beta)	Hazard profile interpretation
Primary CNS Tumor	45 (21–95)	0.85 (0.70-1.02)	Trend toward early-failure tendency
Brain Metastases	38 (18-80)	0.92 (0.81-1.05)	Compatible with early-failure tendency
Non-CNS Solid Tumor	65 (30-120)	1.15 (1.05-1.26)	Increasing hazard tendency

β < 1 indicates early-failure/decreasing hazard tendency; β ≈ 1 indicates approximately constant hazard; β > 1 indicates increasing hazard tendency.

**Table 7:** Seriousness outcomes of inflammatory CNS irAE reports across tumor phenotypes.

Severity outcome	Primary CNS tumor (N = 18)	Brain metastases (N = 95)	Non-CNS solid tumor (N = 410)	P-value
Death	3/18 (16.7%)	17/95 (17.9%)	35/410 (8.5%)	0.045
Life-Threatening	4/18 (22.2%)	24/95 (25.3%)	62/410 (15.1%)	0.012
Hospitalization	14/18 (77.8%)	78/95 (82.1%)	267/410 (65.1%)	<0.001

Notes: Data are presented as n/N (%). The denominator (N) represents the number of patients experiencing CNS inflammatory irAEs within each specific tumor phenotype, not the entire ICI cohort.

### 3.5 JADER shows broadly similar cross-database patterns

In JADER, inflammatory CNS irAE reporting signals showed the same overall direction as FAERS but differed in effect size (**Table 8**). The primary CNS tumor estimate was based on only 4 events among 51 reports and was therefore statistically unstable. Brain metastases included 54 events among 1, 943 reports, yielding an ROR of 1.85 (95% CI, 1.40–2.44).

**Table 8:** Consolidated cross-database comparison of overall, subtype-specific, and multivariable inflammatory CNS irAE reporting analyses.

Analysis domain	Outcome/comparison	FAERS Primary CNS	JADER Primary CNS	FAERS Brain metastases	JADER Brain metastases	Non-CNS reference	Interpretation
Overall disproportionality	Primary narrow inflammatory CNS irAE	Total 2, 145; cases 18; ROR 1.75 (1.08-2.82); IC 0.78 (0.05-1.51)	Total 51; cases 4; ROR 6.07 (2.30-16.01); IC 2.49	Total 5, 630; cases 95; ROR 3.55 (2.85-4.42); IC 1.80 (1.45-2.15)	Total 1, 943; cases 54; ROR 1.85 (1.40-2.44); IC 0.83	FAERS: total 85, 210; cases 410; ROR 1.00 (Ref). JADER: total 56, 873; cases 873; ROR 1.00 (Ref)	Qualitative cross-database agreement; primary CNS estimate unstable because of 4 JADER events; magnitude attenuated in JADER for brain metastases
Key subtype	Encephalitis	Cases 10/ROR 2.10 (1.15-3.85)	Case 4/ROR 8.56 (3.25-22.58)	Case 60/ROR 4.20 (3.10-5.65)	Case 35/ROR 1.68 (1.19-2.36)	Non-CNS solid tumors served as reference in each database	Similar pattern; primary CNS estimates exploratory because of sparse events
Key subtype	Meningitis/aseptic meningitis-related phenotype	Case 5/ROR 0.95 (0.30-3.05)	Case 1/ROR 2.24 (0.14-36.39)	Case 25/ROR 2.85 (1.95-4.15)	Case 19/ROR 2.34 (1.47-3.71)	Non-CNS solid tumors served as reference in each database	Brain metastases showed a similar enrichment pattern; primary CNS estimates unstable
Multivariable association	Primary narrow inflammatory CNS irAE phenotype	Case 18/2, 145; aOR 1.65 (1.02-2.65)	Case 4/51; aOR 5.19 (1.56-12.82)	Case 95/5, 630; aOR 3.12 (2.45-3.98)	Case 54/1, 943; aOR 1.74 (1.30-2.29)	Non-CNS solid tumors served as reference in each database	Similar adjusted pattern; primary CNS exploratory because of sparse JADER events; larger adjusted reporting association in FAERS for brain metastases

Notes: This consolidated table replaces the original Tables 9-11. ROR, reporting odds ratio; IC, information component; aOR, adjusted odds ratio. FAERS was treated as the discovery database and JADER as an independent comparison database. Overall rows report ROR and IC; subtype rows report cases/ROR (95% CI); multivariable rows report cases/total and aOR (95% CI). FAERS multivariable models were adjusted for age, sex, reporting year, reporter type, and treatment regimen; JADER models were adjusted for age, sex, and reporting year. Cross-database interpretation emphasizes qualitative agreement rather than identical effect sizes.

At the subtype level, encephalitis showed the most consistent reporting pattern across databases. Both FAERS and JADER had elevated encephalitis disproportionality signals in primary CNS tumors and brain metastases. By contrast, meningitis/aseptic meningitis-related phenotypes were primarily supported in brain metastases, whereas estimates for primary CNS tumors remained unstable because of very low event counts. Multivariable analyses showed a similar cross-database pattern: for primary CNS tumors relative to non-CNS solid tumors, the aOR was 1.65 (95% CI, 1.02–2.65) in FAERS and 5.19 (95% CI, 1.56–12.82) in JADER; the corresponding aORs for brain metastases were 3.12 (95% CI, 2.45–3.98) and 1.74 (95% CI, 1.30–2.29), respectively (**Table 8**). Taken together, these results support qualitative cross-database agreement, particularly for brain metastases and encephalitis-related phenotypes. However, the primary CNS tumor results in JADER should be regarded as exploratory only.

### 3.6 Parallel single-cell analyses across platforms show immune-compartment enrichment of the strict inflammatory module

**Figure 2:**
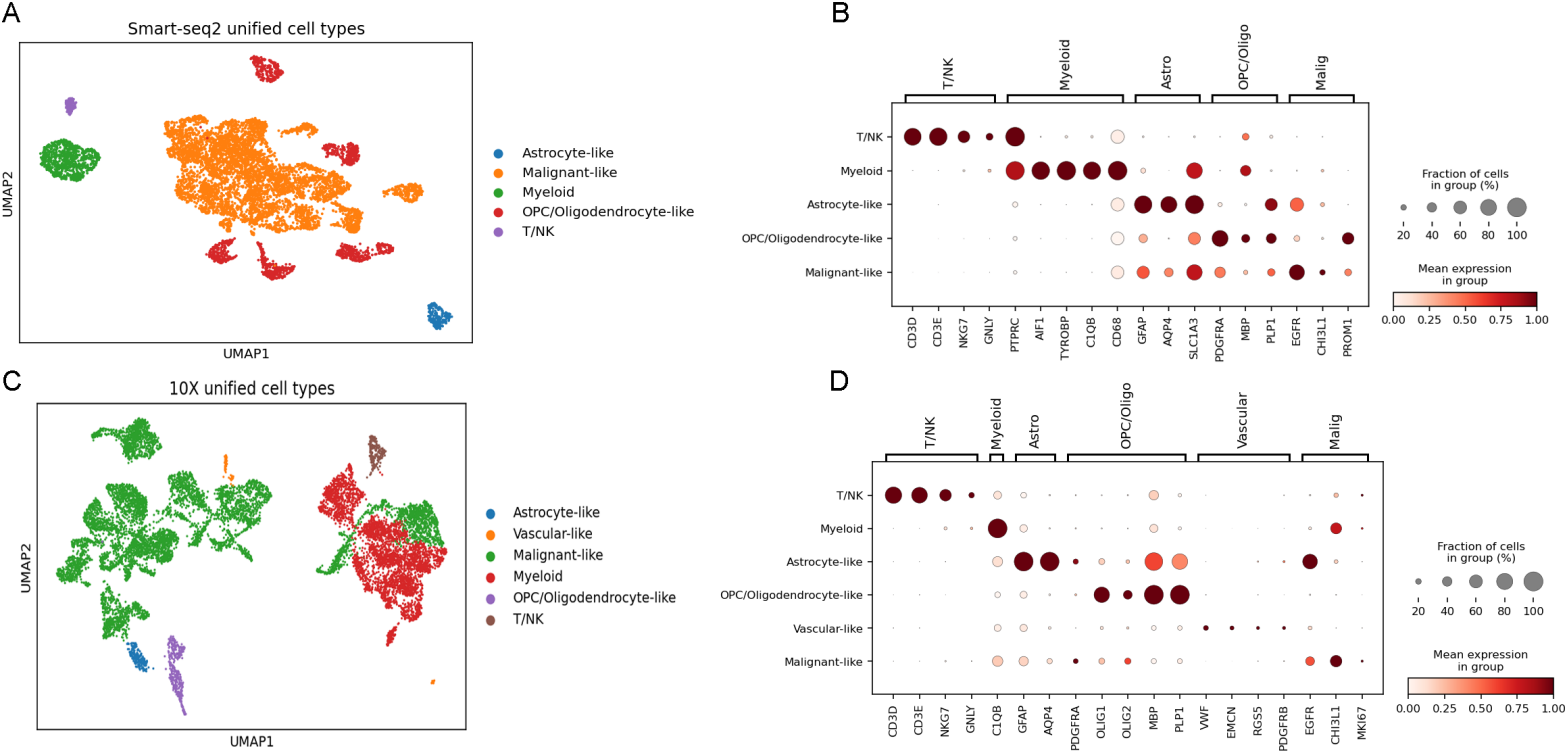
Parallel analysis of Smart-seq2 and 10x GBM single-cell datasets identifies comparable major cellular compartments. **(A)** UMAP visualization of unified cell-type annotations in the GSE131928 Smart-seq2 dataset. Cells were grouped into Astrocyte-like, Malignant-like, Myeloid, OPC/Oligodendrocyte-like, and T/NK compartments. **(B)** Dot plot of representative marker genes supporting the unified cell-type annotations in the Smart-seq2 dataset. Dot size indicates the fraction of cells expressing each marker within a given group, and color intensity indicates the mean normalized expression level. **(C)** UMAP visualization of unified cell-type annotations in the GSE131928 10X dataset. Cells were grouped into Astrocyte-like, Vascular-like, Malignant-like, Myeloid, OPC/Oligodendrocyte-like, and T/NK compartments. **(D)** Dot plot of representative marker genes supporting the unified cell-type annotations in the 10X dataset. Dot size indicates the fraction of cells expressing each marker within a given group, and color intensity indicates the mean normalized expression level.

**Figure 3:**
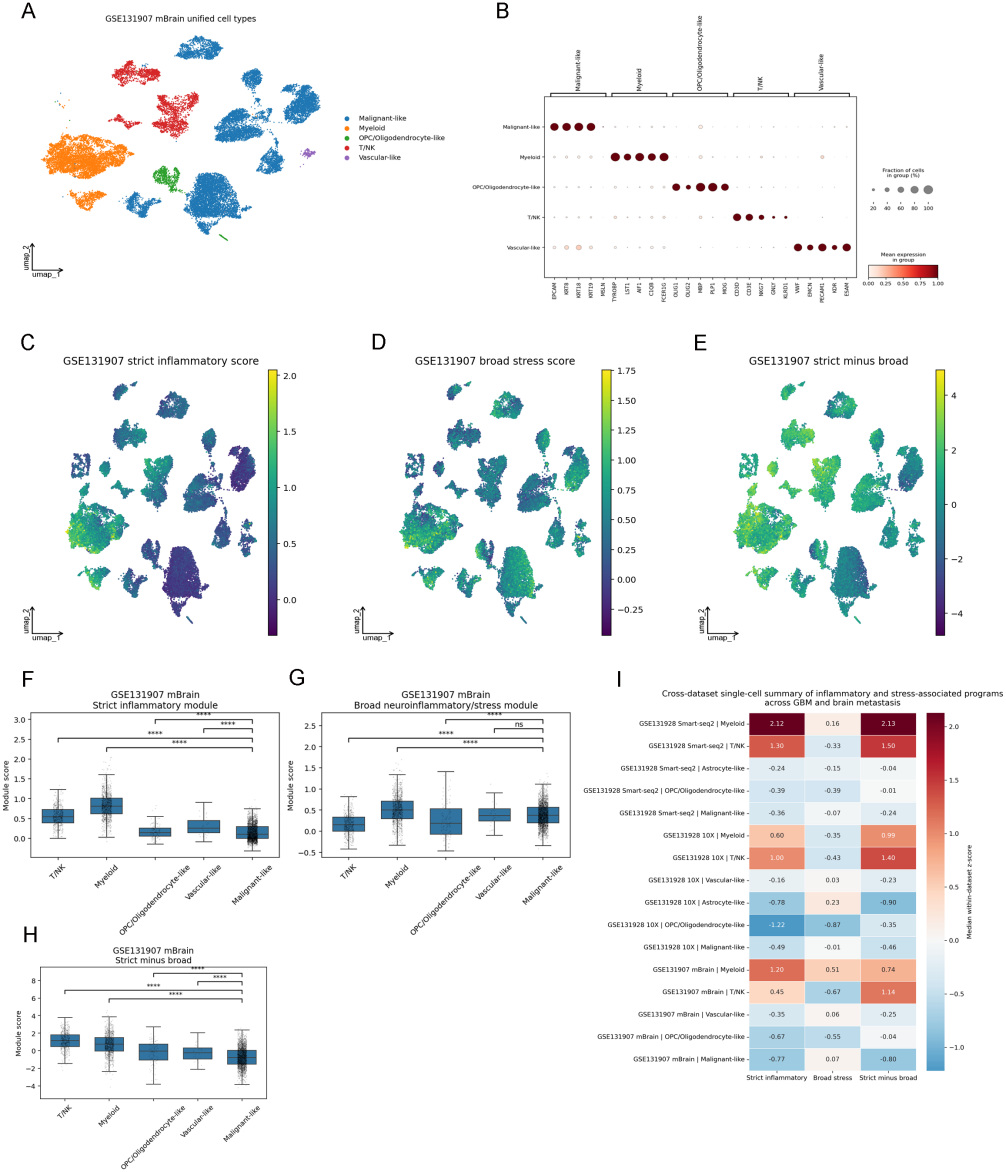
Baseline brain metastasis single-cell analysis and cross-dataset comparison show immune-compartment enrichment of strict inflammatory modules. **(A)** UMAP visualization of unified cell-type annotations in the GSE131907 brain metastasis (mBrain) single-cell dataset. Cells were grouped into Malignant-like, Myeloid, OPC/Oligodendrocyte-like, T/NK, and Vascular-like compartments. **(B)** Dot plot of representative marker genes supporting the unified cell-type annotations in the GSE131907 mBrain dataset. Dot size indicates the fraction of cells expressing each marker within a given group, and color intensity indicates the mean normalized expression level. **(C–E)** UMAP visualization of the strict inflammatory module, broad stress module, and the composite strict-minus-broad score in the GSE131907 mBrain dataset. The strict inflammatory module was preferentially localized to immune-associated compartments, whereas the broad stress module showed a more diffuse distribution across cellular states. **(F–H)** Box plots comparing the strict inflammatory module, broad stress module, and strict-minus-broad score across unified cell compartments in the GSE131907 mBrain dataset. The strict inflammatory module and strict-minus-broad score were highest in Myeloid and T/NK compartments, whereas the broad stress module showed weaker compartment specificity. **(I)** Cross-dataset heatmap summarizing the median within-dataset standardized scores of the strict inflammatory module, broad stress module, and strict-minus-broad score across GSE131928 Smart-seq2, GSE131928 10X, and GSE131907 mBrain datasets. Across datasets, the strict inflammatory module and strict-minus-broad score were consistently elevated in Myeloid and T/NK compartments, whereas the broad stress module lacked comparably stable immune-compartment specificity. Module scores were calculated using predefined gene sets. The strict-minus-broad score was defined as the within-dataset standardized strict inflammatory score minus the standardized broad stress score. Boxes indicate the interquartile range, center lines indicate medians. Statistical comparisons were performed as described in the Methods. ****P < 0.0001.

In parallel analyses of the GSE131928 Smart-seq2, GSE131928 10x, and GSE131907 brain metastasis single-cell datasets, cells were annotated and mapped to harmonized major analytical compartments, including Myeloid, T/NK, Malignant-like, Astrocyte-like or OPC/Oligodendrocyte-like, and Vascular-like compartments where applicable (**Figure 2**; **Figures 3A, B**). Marker gene expression supported the reliability of these harmonized annotations.

**Figure 4:**
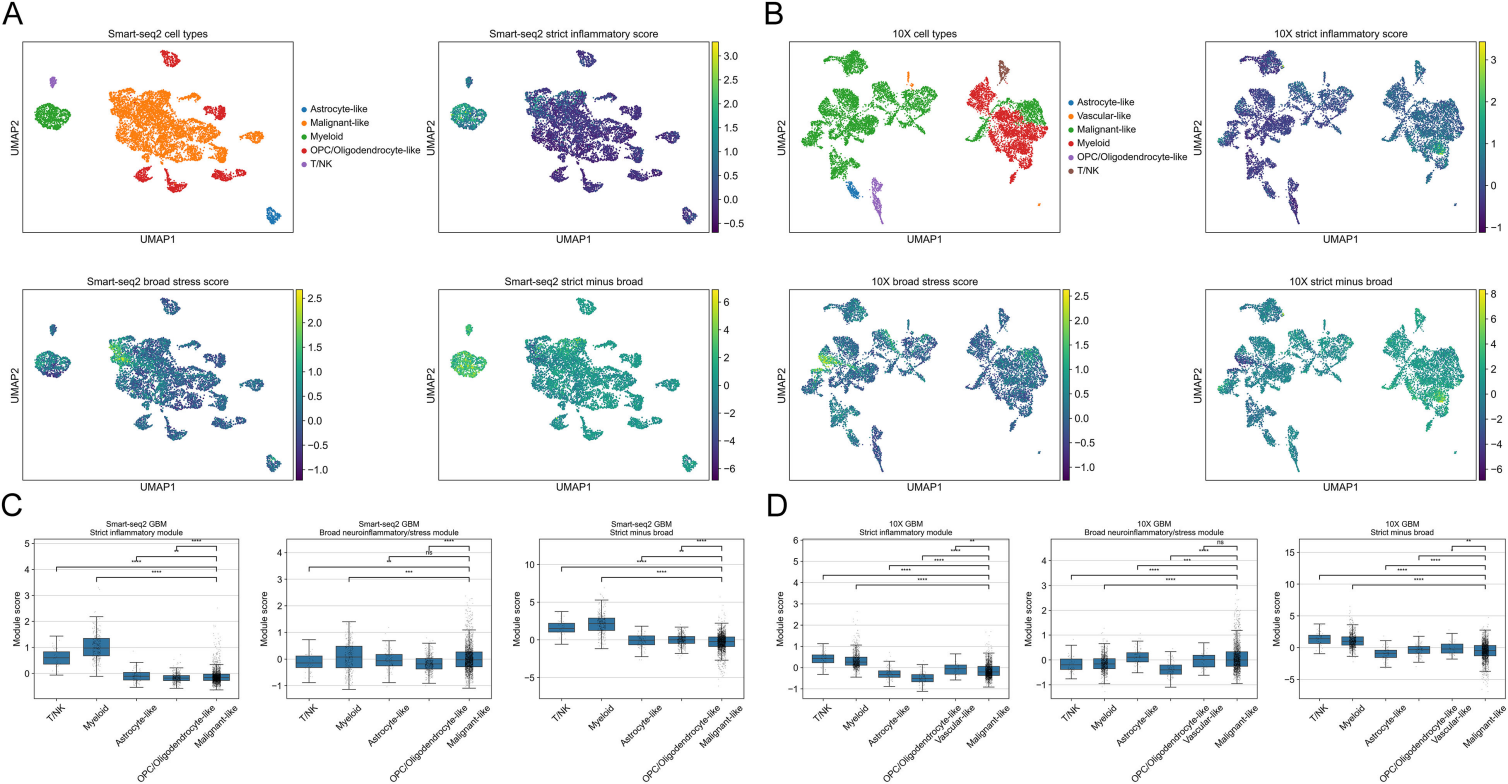
Strict inflammatory modules are enriched in immune cell compartments, whereas broad stress modules are more diffusely distributed across GBM single-cell datasets. **(A)** UMAP visualization of unified cell types and module scores in the GSE131928 Smart-seq2 dataset, including the strict inflammatory module, broad stress module, and the composite strict-minus-broad score. **(B)** UMAP visualization of unified cell types and module scores in the GSE131928 10X dataset, including the strict inflammatory module, broad stress module, and the composite strict-minus-broad score. **(C)** Box plots comparing module scores across unified cell compartments in the Smart-seq2 dataset. The strict inflammatory module was highest in Myeloid and T/NK compartments, whereas the broad stress module showed weaker compartment specificity. The strict-minus-broad score further increased the separation between immune and non-immune compartments. **(D)** Box plots comparing module scores across unified cell compartments in the 10X dataset, showing the same overall pattern as in Smart-seq2. Module scores were calculated using predefined gene sets. The strict-minus-broad score was defined as the within-dataset standardized strict inflammatory score minus the standardized broad stress score. Boxes indicate the interquartile range, center lines indicate medians. Statistical comparisons were performed as described in the Methods. **P < 0.01, ***P < 0.001, ****P < 0.0001.

Module scoring showed a consistent qualitative pattern across datasets. Strict inflammatory scores were enriched mainly in Myeloid and T/NK compartments, whereas broad stress scores were more diffusely distributed across cellular compartments. The strict-minus-broad metric further sharpened the relative separation between immune and non-immune compartments (**Figure 4**; **Figures 3C–I**). Sensitivity analyses using alternative module definitions and residual-based inflammatory scoring preserved the same qualitative compartment-level pattern, with moderate-to-strong correlations with the primary strict-minus-broad metric (Spearman ρ = 0.53–0.93; **Supplementary Figures S1**, **S2**; **Supplementary Table S8**). The lowest correlation was observed in the GSE131928 Smart-seq2 dataset when comparing the primary strict-minus-broad metric with the alternative residual inflammatory score (Spearman ρ = 0.53), indicating only moderate metric-level agreement. However, this did not alter the compartment-level interpretation in that dataset, because the top-scoring compartments remained Myeloid and T/NK.

### 3.7 Secondary descriptive spatial visualization is consistent with immune-enriched module patterns in brain metastases

As a secondary descriptive analysis, we used the GSE179572 brain metastasis Visium dataset to visualize tissue-level representation of the single-cell module patterns; because this dataset included brain metastases only and no formal neighborhood, distance-based, or ligand-receptor inference was performed, it was not treated as an independent evidence layer.

In representative brain metastasis samples, strict inflammatory and strict-minus-broad signals appeared in focal tissue regions that overlapped with immune-rich and myeloid-like spatial signatures, whereas broad stress signals were more diffusely distributed (**Supplementary Figures S3**–**S5**). This pattern was consistent with the single-cell observation that strict inflammatory module activity was carried primarily by immune-related compartments. However, because inflammatory modules are expected to colocalize with immune-cell-enriched regions, these spatial maps should be interpreted only as descriptive tissue-level visualization of the single-cell-derived module pattern, rather than as independent mechanistic evidence or confirmation of irAE-specific biology.

## 4 Discussion

This study combined FAERS signal discovery, JADER cross-database comparison, and transcriptomic contextualization to evaluate inflammatory CNS irAE reporting across brain tumor phenotypes. Overall, reporting signals were most prominent in brain metastases, attenuated under broader neuroinflammatory definitions, and not reproduced by thyroid or myocarditis-related comparator phenotypes. JADER showed a broadly similar pattern, although primary CNS tumor estimates remained exploratory because of sparse event counts. Single-cell analyses localized the strict inflammatory module mainly to myeloid and T/NK compartments, whereas spatial maps served only as secondary descriptive visualization. Together, these findings support a hypothesis-generating framework in which stricter inflammatory CNS irAE phenotypes show tumor phenotype-associated reporting heterogeneity, while pharmacovigilance and transcriptomic data remain non-causal.

Surveillance and detection bias deserve particular consideration in this study. Patients with brain metastases are more likely to undergo repeated brain MRI, neurologic examinations, corticosteroid or anti-edema management, hospitalization, and multidisciplinary neurologic or oncologic assessment than patients with non-CNS solid tumors. These differences may increase the likelihood that neurologic symptoms, radiographic changes, or suspected CNS inflammatory events are recognized, coded, and submitted to spontaneous reporting systems. Therefore, the more prominent inflammatory CNS irAE reporting signal observed in brain metastases may partly reflect differential detection and reporting intensity rather than a true increase in clinical risk. The negative-control thyroid irAE analysis helped assess whether brain tumor-related cohorts had globally increased irAE reporting, but thyroid events are typically detected through laboratory monitoring and do not fully address neurologic surveillance bias. We therefore added a myocarditis-related non-CNS serious irAE comparator, which requires clinical suspicion and diagnostic work-up. The myocarditis-related comparator was retained only as a descriptive diagnostic-workup comparator. However, because the brain metastasis subgroup contained only one myocarditis event, the corresponding estimate was severely underpowered and statistically uninformative, and should not be interpreted as evidence against surveillance bias. Nevertheless, even this comparator cannot fully reproduce the surveillance structure of CNS inflammatory events; thus, residual CNS-specific detection bias remains an important limitation.

The brain metastasis reporting pattern should be interpreted cautiously. Although inflammatory CNS irAE reporting signals were more prominent in brain metastases than in primary CNS tumors across several analyses, residual differences in neurologic surveillance, hospitalization, and reporting opportunity may have contributed to this pattern. This observation is compatible with, but does not prove, the view that brain tumor backgrounds represent a spectrum of immune microenvironmental states rather than a single immunologic entity, with primary CNS tumors such as GBM often showing immunosuppressive myeloid-enriched niches and brain metastases reflecting a complex tumor-host interface shaped by CNS inflammation, vascular responses, and myeloid remodeling (24–30). Conversely, primary CNS tumor findings were based on limited FAERS events and extremely sparse JADER events, and should therefore be regarded as exploratory, hypothesis-generating reporting signals rather than established associations.

The divergence between strict and broad phenotype definitions suggests that outcome refinement influenced the specificity of the reporting phenotype (31). Broad neuroinflammatory definitions may capture cerebral edema, hypoxia, treatment-related injury, metabolic disturbance, or tumor progression, whereas stricter definitions are more likely to approximate immune-mediated CNS inflammation. The absence of comparable enrichment in thyroid and myocarditis-related comparator analyses further argues against a purely generalized irAE reporting pattern, although CNS-specific surveillance bias remains possible.

Subtype- and regimen-stratified analyses aligned with the primary findings. Encephalitis appeared to be the most information-rich subtype, whereas meningitis- and myelitis-related estimates were constrained by sparse counts, particularly in primary CNS tumors. Combination therapy showed more prominent reporting signals, consistent with the broader clinical understanding that more intense immune activation may be accompanied by greater toxicity (32–34). These findings remain reporting associations and should not be interpreted as mechanistic evidence.

From a clinical perspective, these findings may help refine vigilance strategies rather than directly determine ICI eligibility. For patients with brain metastases or primary CNS tumors receiving ICIs, especially those treated with combination regimens, clinicians may consider heightened baseline neurologic assessment, careful documentation of pre-existing neurologic symptoms, and closer monitoring for early symptoms compatible with encephalitis, meningitis, or myelitis (6, 13). Because tumor progression, radiation injury, infection, edema, metabolic disturbances, and ICI-related inflammation can present with overlapping neurologic or radiographic features, suspected CNS irAEs should prompt timely multidisciplinary evaluation involving oncology, neurology, radiology, and infectious disease teams when appropriate. The present findings also support the need for structured reporting and adjudication of neurologic irAEs in brain tumor populations. However, because FAERS and JADER cannot estimate incidence or prove causality, these results should be used to inform risk awareness and surveillance priorities, not to withhold or mandate ICI treatment.

The single-cell results should be interpreted as baseline contextualization rather than biological validation of irAE mechanisms. Across primary GBM and brain metastasis datasets, the strict inflammatory module was enriched mainly in myeloid and T/NK compartments, whereas the broad stress module was more diffusely distributed. Myeloid compartments showed the most stable high scores, consistent with their recognized role as major immune components of brain tumor microenvironments, but this does not establish that myeloid cells directly mediate CNS irAEs after ICI exposure (35). The strict inflammatory and broad stress modules, as well as the strict-minus-broad metric, remain predefined exploratory constructs rather than irAE-specific signatures or validated biomarkers. Sensitivity analyses using alternative module definitions and residual-based scoring preserved the same qualitative compartment-level pattern, but tissues from clinically adjudicated CNS irAE cases will be required to define irAE-specific transcriptional programs.

Within this exploratory framework, the strict inflammatory module mainly captured IFN/interferon-stimulated signaling, antigen presentation, inflammatory chemokine expression, TNF/NF-κB-related activation, and adhesion-related programs (19). Its enrichment in myeloid and T/NK compartments may therefore reflect pre-existing immune-inflammatory niches that are more capable of antigen presentation, immune-cell recruitment, and local inflammatory amplification. By contrast, the broad stress module captured hypoxia, angiogenic/metabolic stress, immediate early stress responses, and heat-shock or injury-associated programs, which are less specific to immune-mediated inflammation and may also reflect tumor stress, edema, hypoxia, or treatment-related injury (21). Thus, the contrast between these modules provides a hypothesis-generating framework for distinguishing inflammation-dominant immune niches from nonspecific stress-dominant tissue states. However, because these datasets were not obtained from patients with CNS irAEs, these patterns should not be interpreted as causal evidence linking either module to ICI-related CNS irAEs.

The spatial transcriptomic component should be interpreted as a secondary descriptive visualization rather than as a separate mechanistic evidence layer. We used the brain metastasis-only Visium dataset GSE179572 to examine whether the single-cell-derived immune-compartment module pattern could also be visualized in tissue space. As expected, strict inflammatory and strict-minus-broad signals overlapped with immune-rich and myeloid-like regions, whereas broad stress signals appeared more diffuse. This observation is consistent with the single-cell results, but it is not independent proof of a distinct spatial mechanism because inflammatory gene modules are partly defined by genes expressed in immune cells and are therefore expected to colocalize with immune-enriched regions (36). Moreover, GSE179572 does not include primary GBM spatial samples, and we did not perform formal spatial-neighborhood, distance-based, or ligand-receptor communication analyses. Accordingly, the spatial maps should be viewed as supportive visualization of baseline tissue organization in brain metastases, not as a separate evidence layer or validation of irAE-specific biology.

This study has several limitations. First, FAERS and JADER are spontaneous reporting databases and are therefore subject to underreporting, incomplete clinical information, duplicate or stimulated reporting, heterogeneous coding practices, and differences in reporting behavior across countries and healthcare systems. Because these databases lack true exposure denominators, standardized follow-up, and strict clinical adjudication, the observed RORs and adjusted reporting associations should be interpreted as disproportionality signals rather than estimates of incidence, absolute risk, or causal effects. These limitations may influence effect-size estimates and partly explain between-database heterogeneity; therefore, the FAERS-JADER comparison was used to assess qualitative agreement in reporting patterns, not to validate identical effect sizes or true clinical risk. Second, surveillance, detection, and residual confounding remain important limitations. Patients with brain metastases and primary CNS tumors undergo more intensive neurologic monitoring, neuroimaging, healthcare contact, and hospitalization than patients with non-CNS solid tumors, which may increase the likelihood that neurologic symptoms or radiographic abnormalities are recognized and reported as suspected CNS irAEs. Thus, the more prominent reporting signal observed in brain metastases may partly reflect detection intensity or reporting opportunity rather than true increased clinical risk. Although the thyroid negative-control analysis helped assess generalized irAE reporting, and the myocarditis-related non-CNS serious irAE comparator was added as a diagnostic-workup comparator, neither can account for CNS-specific surveillance bias; in particular, the myocarditis comparator was underpowered and statistically uninformative for the brain metastasis subgroup because it included only one event. Moreover, disease stage, baseline neurologic status, prior radiotherapy, corticosteroid use, and detailed concomitant medications could not be fully adjusted for because these variables were incompletely or inconsistently recorded in FAERS and JADER. Third, event counts for primary CNS tumors were limited, with only 18 inflammatory CNS irAE events in FAERS and 4 events in JADER. Subtype analyses were even more sparse, including 10 encephalitis, 5 meningitis, and 3 myelitis-related events in FAERS. Accordingly, all primary CNS tumor subgroup findings should be interpreted as exploratory and hypothesis-generating rather than as established associations, and the JADER primary CNS tumor analysis should be viewed as descriptive cross-database comparison rather than statistical validation. Fourth, the public single-cell and spatial transcriptomic datasets were not obtained from patients with clinically adjudicated CNS irAEs or from tissues sampled at irAE onset. These analyses therefore cannot demonstrate irAE-specific cellular mechanisms or directly explain differential CNS irAE reporting across tumor backgrounds. They should instead be interpreted as descriptive characterization of pre-existing immune and stress-related landscapes. The spatial transcriptomic component was intentionally reduced to secondary descriptive visualization because the available dataset included brain metastases only, lacked primary GBM spatial samples, and did not include formal spatial-neighborhood, distance-based, or ligand-receptor analyses. Fifth, the strict inflammatory and broad stress modules, as well as the strict-minus-broad metric, are predefined exploratory constructs rather than validated irAE-specific biomarkers. Although sensitivity analyses supported similar qualitative compartment-level patterns, alternative module definitions may yield different quantitative values. In addition, the single-cell datasets differed in sequencing platform and sample composition. Larger uniformly generated single-cell and spatial cohorts, ideally including tissues collected at or near CNS irAE onset, will be required to improve reproducibility and test mechanistic hypotheses more directly.

In summary, inflammatory CNS irAE reporting signals suggested tumor phenotype-associated heterogeneity after ICI exposure, with the most prominent disproportionality signals observed in brain metastases. Stricter inflammatory definitions appeared more specific than broad neuroinflammatory definitions, while single-cell transcriptomic analyses provided baseline contextualization of immune-enriched myeloid/T/NK compartments. These findings should be interpreted as hypothesis-generating reporting associations rather than estimates of incidence, risk, causality, or irAE-specific tissue biology. Prospective clinically adjudicated cohorts with tissue sampled at or near irAE onset will be required to test these hypotheses directly.

## Data Availability

The datasets presented in this study can be found in online repositories. The names of the repository/repositories and accession number(s) can be found below: https://www.ncbi.nlm.nih.gov/, GSE131928 https://www.ncbi.nlm.nih.gov/, GSE131907 https://www.ncbi.nlm.nih.gov/, GSE179572 https://fis.fda.gov/extensions/FPD-QDE-FAERS/FPD-QDE-FAERS.html, N/A https://www.pmda.go.jp/safety/info-services/drugs/adr-info/suspected-adr/0005.html, N/A.

## Supplementary material

Supplementary Figure 1 Sensitivity analyses using alternative module definitions and residual-based relative inflammatory scoring in GSE131928. **(A)** Compartment-level median standardized scores in the GSE131928 Smart-seq2 dataset across primary module scores, primary strict-minus-broad score, primary residual inflammatory score, alternative module scores, alternative minus-stress score, and alternative residual inflammatory score. **(B)** Corresponding compartment-level median standardized scores in the GSE131928 10x dataset. **(C)** Spearman correlations between the primary strict-minus-broad metric and sensitivity metrics, including primary residual inflammatory score, alternative minus-stress score, and alternative residual inflammatory score. These sensitivity analyses were used to evaluate whether the main immune-compartment enrichment pattern depended on the primary gene lists or the subtraction-based composite metric.

Supplementary Figure 2 Sensitivity analyses using alternative module definitions and residual-based relative inflammatory scoring in GSE131907 mBrain. **(A)** Compartment-level median standardized scores across primary module scores, primary strict-minus-broad score, primary residual inflammatory score, alternative module scores, alternative minus-stress score, and alternative residual inflammatory score. **(B)** Spearman correlations between the primary strict-minus-broad metric and sensitivity metrics, including primary residual inflammatory score, alternative minus-stress score, and alternative residual inflammatory score. These analyses were used to evaluate whether the main immune-compartment enrichment pattern depended on the primary gene lists or the subtraction-based composite metric.

Supplementary Figure 3 Representative baseline spatial maps show focal immune-enriched niches with elevated strict inflammatory module activity in brain metastases. Spatial maps from brain metastasis samples pt15 and pt27 showing the strict inflammatory module, broad stress module, strict-minus-broad score, and myeloid-like, T/NK-like, and tumor-like signatures. Strict inflammatory and strict-minus-broad signals were most closely aligned with myeloid-like niches, whereas broad stress signals were more diffuse.

Supplementary Figure 4 Continuous baseline spatial maps across all analyzed brain metastasis samples. Spatial distributions of the strict inflammatory module, broad stress module, strict-minus-broad score, and compartment-related signatures across all analyzed samples.

Supplementary Figure 5 Simplified niche annotations across all analyzed brain metastasis samples. Simplified niche annotations showing immune-rich, tumor-rich, vascular-like, oligo-like, mixed, and low-signal regions across all analyzed samples.

## References

[1] Sharma P Allison JP . The future of immune checkpoint therapy. Sci (New York NY). (2015) 348:56–61. doi: 10.1126/science.aaa8172 25838373

[2] Grippin AJ Marconi C Copling S Li N Braun C Woody C . SARS-CoV-2 mRNA vaccines sensitize tumours to immune checkpoint blockade. (2025) 647:488–97. doi: 10.1038/s41586-025-09655-y PMC12611756 41125896

[3] Postow MA Sidlow R Hellmann MD . Immune-related adverse events associated with immune checkpoint blockade. N Engl J Med. (2018) 378:158–68. doi: 10.1056/NEJMra1703481 29320654

[4] Cuzzubbo S Javeri F Tissier M Roumi A Barlog C Doridam J . Neurological adverse events associated with immune checkpoint inhibitors: Review of the literature. Eur J Cancer (Oxford Engl 1990). (2017) 73:1–8. doi: 10.1016/j.ejca.2016.12.001 28064139

[5] Dubey D David WS Reynolds KL Chute DF Clement NF Cohen JV . Severe neurological toxicity of immune checkpoint inhibitors: Growing spectrum. Ann Neurol. (2020) 87:659–69. doi: 10.1002/ana.25708 32086972

[6] Haanen J Obeid M Spain L Carbonnel F Wang Y Robert C . Management of toxicities from immunotherapy: ESMO Clinical Practice Guideline for diagnosis, treatment and follow-up. Ann Oncol Off J Eur Soc For Med Oncol. (2022) 33:1217–38. doi: 10.1016/j.annonc.2022.10.001 36270461

[7] Klemm F Maas RR Bowman RL Kornete M Soukup K Nassiri S . Interrogation of the microenvironmental landscape in brain tumors reveals disease-specific alterations of immune cells. Cell. (2020) 181:1643–60. doi: 10.1016/j.cell.2020.05.007 32470396 PMC8558904

[8] Lin H Liu C Hu A Zhang D Yang H Mao Y . Understanding the immunosuppressive microenvironment of glioma: Mechanistic insights and clinical perspectives. J Hematol Oncol. (2024) 17:31. doi: 10.1186/s13045-024-01544-7 38720342 PMC11077829

[9] Dong W Sheng J Cui JZM Zhao H Wong STC . Systems immunology insights into brain metastasis. Trends Immunol. (2024) 45:903–16. doi: 10.1016/j.it.2024.09.010 39443266 PMC12049182

[10] Karimi E Yu MW . Single-cell spatial immune landscapes of primary and metastatic brain tumours. (2023) 614:555–63. doi: 10.1038/s41586-022-05680-3 PMC9931580 36725935

[11] Roth P Winklhofer S Müller AMS Dummer R Mair MJ Gramatzki D . Neurological complications of cancer immunotherapy. Cancer Treat Rev. (2021) 97:102189. doi: 10.1016/j.ctrv.2021.102189 33872978

[12] Guidon AC Burton LB Chwalisz BK Hillis J Schaller TH Amato AA . Consensus disease definitions for neurologic immune-related adverse events of immune checkpoint inhibitors. (2021) 9:1. doi: 10.1136/jitc-2021-002890 PMC8291304 34281989

[13] Willis MD Schroeder B Marandino L Turajlic S Carr AS . Neurological immune-related adverse events with checkpoint inhibitor therapy: Challenges for the neurologist. J Neurology Neurosurgery Psychiatry. (2025) 96:1024–37. doi: 10.1136/jnnp-2025-335998 40675801 PMC12573370

[14] Lou S Chen H Cui Z Zhang X Zhu C Zhou L . Safety evaluation of irinotecan: A real-world disproportionality analysis using FAERS and JADER databases during the time period 2004-2024. Front Pharmacol. (2025) 16:1516449. doi: 10.3389/fphar.2025.1516449 40552159 PMC12184384

[15] Neftel C Laffy J Filbin MG Hara T Shore ME Rahme GJ . An integrative model of cellular states, plasticity, and genetics for glioblastoma. Cell. (2019) 178:835–849.e821. doi: 10.1016/j.cell.2019.06.024 31327527 PMC6703186

[16] Kim N Kim HK Lee K . Single-cell RNA sequencing demonstrates the molecular and cellular reprogramming of metastatic lung adenocarcinoma. (2020) 11:2285. doi: 10.1038/s41467-020-16164-1 PMC7210975 32385277

[17] Subramanian A Tamayo P Mootha VK Mukherjee S Ebert BL Gillette MA . Gene set enrichment analysis: A knowledge-based approach for interpreting genome-wide expression profiles. PNAS. (2005) 102:15545–50. doi: 10.1073/pnas.0506580102 16199517 PMC1239896

[18] Liberzon A Birger C Thorvaldsdóttir H Ghandi M Mesirov JP Tamayo P . The Molecular Signatures Database (MSigDB) hallmark gene set collection. Cell Syst. (2015) 1:417–25. doi: 10.1016/j.cels.2015.12.004 26771021 PMC4707969

[19] Ivashkiv LB . IFNγ: Signalling, epigenetics and roles in immunity, metabolism, disease and cancer immunotherapy. Nat Rev Immunol. (2018) 18:545–58. doi: 10.1038/s41577-018-0029-z 29921905 PMC6340644

[20] Dangaj D Bruand M Grimm AJ Ronet C Barras D Duttagupta PA . Cooperation between constitutive and inducible chemokines enables T cell engraftment and immune attack in solid tumors. Cancer Cell. (2019) 35:885–900. doi: 10.1016/j.ccell.2019.05.004 31185212 PMC6961655

[21] Dengler VL Galbraith M Espinosa JM . Transcriptional regulation by hypoxia inducible factors. Crit Rev Biochem Mol Biol. (2014) 49:1–15. doi: 10.3109/10409238.2013.838205 24099156 PMC4342852

[22] Hai T Wolford CC Chang YS . ATF3, a hub of the cellular adaptive-response network, in the pathogenesis of diseases: Is modulation of inflammation a unifying component? Gene Expression. (2010) 15:1–11. doi: 10.3727/105221610x12819686555015 21061913 PMC6043823

[23] Sudmeier LJ Hoang KB Nduom EK Wieland A Neill SG Schniederjan MJ . Distinct phenotypic states and spatial distribution of CD8(+) T cell clonotypes in human brain metastases. Cell Rep Med. (2022) 3:100620. doi: 10.1016/j.xcrm.2022.100620 35584630 PMC9133402

[24] Jiang J Wu L Yuan F Ji J Lin X Yang W . Characterization of the immune microenvironment in brain metastases from different solid tumors. (2020) 9:2299–308. doi: 10.1002/cam4.2905 PMC7131856 32017467

[25] De Leo A Ugolini A . Myeloid cells in glioblastoma microenvironment. (2020) 10:18. doi: 10.3390/cells10010018 PMC7824606 33374253

[26] Schreurs LD Vom Stein AF Jünger ST Timmer M Noh KW Buettner R . The immune landscape in brain metastasis. (2025) 27:50–62. doi: 10.1093/neuonc/noae219 PMC11726252 39403738

[27] Feng Y Hu X Zhang Y Wang Y . The role of microglia in brain metastases: Mechanisms and strategies. Aging Dis. (2024) 15:169–85. doi: 10.14336/ad.2023.0514 37307835 PMC10796095

[28] Wu Y Sun R Ren S Zengin G Li M . Neuronal reshaping of the tumor microenvironment in tumorigenesis and metastasis: Bench to clinic. Med Adv. (2025) 3:364–71. doi: 10.1002/med4.70044 41531421

[29] Li M-Y Zhang Q Zengin G Guo Q Sun W . Spatial omics: Deciphering heterogeneity in the tumor immune microenvironment and resistance to immunotherapy. Curr Proteomics. (2025) 22:100055. doi: 10.1016/j.curpro.2025.100055 38826717

[30] Che X Wang H Han W Jiang YZ . Intratumoral nerve phased development: A promising therapeutic target. Med Bull. (2025) 1:154–170. doi: 10.1002/mdb2.70009 41531421

[31] Naidoo J Murphy C Atkins MB . Society for Immunotherapy of Cancer (SITC) consensus definitions for immune checkpoint inhibitor-associated immune-related adverse events (irAEs) terminology. (2023) 11:e006398. doi: 10.1136/jitc-2022-006398 PMC10069596 37001909

[32] Buckley MW Balaji Warner A . Immune-related encephalitis after immune checkpoint inhibitor therapy. (2025) 30:oyae186. doi: 10.1093/oncolo/oyae186 PMC11783331 39066587

[33] Farina A Birzu C Elsensohn MH Picca A . Neurological outcomes in immune checkpoint inhibitor-related neurotoxicity. (2023) 5:. doi: 10.1093/braincomms/fcad169 PMC10306160 37389303

[34] Yin Q Wu L Han L Zheng X Tong R Li L . Immune-related adverse events of immune checkpoint inhibitors: A review. Front Immunol. (2023) 14:1167975. doi: 10.3389/fimmu.2023.1167975 37304306 PMC10247998

[35] Huang B Zhang J Zong W Chen S Zong Z Zeng X . Myeloidcells in the immunosuppressive microenvironment in glioblastoma: The characteristics and therapeutic strategies. Front Immunol. (2023) 14:994698. doi: 10.3389/fimmu.2023.994698 36923402 PMC10008967

[36] Christensson G Bocci M . Spatial multiomics reveals intratumoral immune heterogeneity with distinct cytokine networks in lung cancer brain metastases. (2024) 4:2888–902. doi: 10.1158/2767-9764.crc-24-0201 PMC11539001 39400127

