# Supplementary material for: Heterogeneity of immune checkpoint inhibitor-related inflammatory central nervous system adverse event reporting signals in primary and metastatic brain tumors: a pharmacovigilance study with single-cell and spatial transcriptomic contextualization
Source: Front Immunol. 2026 Jul 8;17:1866830. doi: 10.3389/fimmu.2026.1866830 (PMC13388250; doi:10.3389/fimmu.2026.1866830)
Supplement: Supplementary Figure 1 — Sensitivity analyses using alternative module definitions and residual-based relative inflammatory scoring in GSE131928. (A) Compartment-level median standardized scores in the GSE131928 Smart-seq2 dataset across primary module scores, primary strict-minus-broad score, primary residual inflammatory score, alternative module scores, alternative minus-stress score, and alternative residual inflammatory score. (B) Corresponding compartment-level median standardized scores in the GSE131928 10x dataset. (C) Spearman correlations between the primary strict-minus-broad metric and sensitivity metrics, including primary residual inflammatory score, alternative minus-stress score, and alternative residual inflammatory score. These sensitivity analyses were used to evaluate whether the main immune-compartment enrichment pattern depended on the primary gene lists or the subtraction-based composite metric. [file Table1.docx]

| **Table S1. Data-processing attrition table.** | |
| --- | --- |
| Data Processing Step | Remaining Reports (N) |
| Total adverse event reports in FAERS (Jan 2014 - Dec 2025) | 15,450,210 |
| Exclusion: Non-ICI suspicious drug exposure | 450,120 |
| Exclusion: Duplicate reports based on CASEID and FDA_DT | 235,450 |
| Subcohort: Patients with identified primary CNS tumors | 2,145 |
| Subcohort: Patients with identified brain metastases | 5,630 |
| Subcohort: Patients with identified non-CNS solid tumors | 85,210 |
| Final Analysis Cohort | 92,985 |
| Notes: Depicts the step-by-step cohort selection process. | |
