# Supplementary material for: Heterogeneity of immune checkpoint inhibitor-related inflammatory central nervous system adverse event reporting signals in primary and metastatic brain tumors: a pharmacovigilance study with single-cell and spatial transcriptomic contextualization
Source: Front Immunol. 2026 Jul 8;17:1866830. doi: 10.3389/fimmu.2026.1866830 (PMC13388250; doi:10.3389/fimmu.2026.1866830)
Supplement: Supplementary Figure 2 — Sensitivity analyses using alternative module definitions and residual-based relative inflammatory scoring in GSE131907 mBrain. (A) Compartment-level median standardized scores across primary module scores, primary strict-minus-broad score, primary residual inflammatory score, alternative module scores, alternative minus-stress score, and alternative residual inflammatory score. (B) Spearman correlations between the primary strict-minus-broad metric and sensitivity metrics, including primary residual inflammatory score, alternative minus-stress score, and alternative residual inflammatory score. These analyses were used to evaluate whether the main immune-compartment enrichment pattern depended on the primary gene lists or the subtraction-based composite metric. [file Table2.docx]

| **Table S2. Multivariable regression full model output.** | | | |
| --- | --- | --- | --- |
| Variable | Adjusted OR (aOR) | 95% CI | p-value |
| Tumor: Brain Metastases (vs Non-CNS) | 3.12 | 2.45 - 3.98 | <0.001 |
| Tumor: Primary CNS (vs Non-CNS) | 1.65 | 1.02 - 2.65 | 0.041 |
| Age >= 65 (vs <65) | 1.15 | 0.95 - 1.39 | 0.155 |
| Sex: Male (vs Female) | 1.02 | 0.85 - 1.22 | 0.840 |
| Reporter: HCP (vs Non-HCP) | 1.25 | 1.05 - 1.48 | 0.012 |
| Regimen: Combination (vs Monotherapy) | 2.10 | 1.85 - 2.38 | <0.001 |
| Reporting Year (Continuous) | 1.08 | 1.01 - 1.15 | 0.024 |
| Notes: Baseline reference groups are omitted from the table (e.g., Age < 65, Female). | | | |
