# Supplementary material for: Heterogeneity of immune checkpoint inhibitor-related inflammatory central nervous system adverse event reporting signals in primary and metastatic brain tumors: a pharmacovigilance study with single-cell and spatial transcriptomic contextualization
Source: Front Immunol. 2026 Jul 8;17:1866830. doi: 10.3389/fimmu.2026.1866830 (PMC13388250; doi:10.3389/fimmu.2026.1866830)
Supplement: Supplementary Figure 3 — Representative baseline spatial maps show focal immune-enriched niches with elevated strict inflammatory module activity in brain metastases. Spatial maps from brain metastasis samples pt15 and pt27 showing the strict inflammatory module, broad stress module, strict-minus-broad score, and myeloid-like, T/NK-like, and tumor-like signatures. Strict inflammatory and strict-minus-broad signals were most closely aligned with myeloid-like niches, whereas broad stress signals were more diffuse. [file Table3.docx]

| **Table S3. Sensitivity analyses beyond HCP-only.** | | | |
| --- | --- | --- | --- |
| Sensitivity Model | Primary CNS: aOR (95% CI) | Brain Mets: aOR (95% CI) | Conclusion |
| 1. Suspect Drug (PS) Role Only | 1.65 (1.02-2.65) | 3.12 (2.45-3.98) | Main model baseline |
| 2. PS + Concomitant (SS) Roles | 1.52 (0.95-2.45) | 2.85 (2.20-3.65) | Diluted but consistent |
| 3. Excluding concurrent Systemic Corticosteroids | 1.85 (1.10-3.15) | 3.45 (2.65-4.55) | Signal strengthened |
| 4. Excluding concurrent Bevacizumab/TMZ exposure | 1.68 (1.05-2.70) | 3.10 (2.40-3.95) | Maintained |
| Notes: SS, Secondary Suspect; TMZ, Temozolomide. | | | |
