# Supplementary material for: Heterogeneity of immune checkpoint inhibitor-related inflammatory central nervous system adverse event reporting signals in primary and metastatic brain tumors: a pharmacovigilance study with single-cell and spatial transcriptomic contextualization
Source: Front Immunol. 2026 Jul 8;17:1866830. doi: 10.3389/fimmu.2026.1866830 (PMC13388250; doi:10.3389/fimmu.2026.1866830)
Supplement: Supplementary Figure 4 — Continuous baseline spatial maps across all analyzed brain metastasis samples. Spatial distributions of the strict inflammatory module, broad stress module, strict-minus-broad score, and compartment-related signatures across all analyzed samples. [file Table4.docx]

| **Table S4. Descriptive subtype-specific reporting profiles of inflammatory CNS irAEs.** | | | |
| --- | --- | --- | --- |
| irAE Subtype | Primary CNS Tumor: n, ROR (95% CI) | Brain Metastases: n, ROR (95% CI) | Non-CNS Solid Tumor (Ref): n |
| Encephalitis | 10, 2.10 (1.15-3.85) | 60, 4.20 (3.10-5.65) | 220 |
| Meningitis | 5, 0.95 (0.30-3.05) | 25, 2.85 (1.95-4.15) | 140 |
| Myelitis | 3, 1.10 (0.45-2.65) | 10, 1.55 (0.85-2.85) | 50 |
| Notes: Event counts and RORs are presented descriptively. Primary CNS tumor subtype analyses were underpowered because of very small event counts, including 10 encephalitis, 5 meningitis, and 3 myelitis-related events. These estimates should not be interpreted as established subtype-specific associations. | | | |
