# Supplementary material for: Heterogeneity of immune checkpoint inhibitor-related inflammatory central nervous system adverse event reporting signals in primary and metastatic brain tumors: a pharmacovigilance study with single-cell and spatial transcriptomic contextualization
Source: Front Immunol. 2026 Jul 8;17:1866830. doi: 10.3389/fimmu.2026.1866830 (PMC13388250; doi:10.3389/fimmu.2026.1866830)
Supplement: Supplementary Figure 5 — Simplified niche annotations across all analyzed brain metastasis samples. Simplified niche annotations showing immune-rich, tumor-rich, vascular-like, oligo-like, mixed, and low-signal regions across all analyzed samples. [file Table5.docx]

| **Table S5. Detailed PT frequencies within inflammatory CNS irAE cases.** | | | |
| --- | --- | --- | --- |
| MedDRA PT | Primary CNS (n) | Brain Metastases (n) | Non-CNS Solid Tumor (n) |
| Encephalitis | 7 | 45 | 160 |
| Autoimmune encephalitis | 3 | 15 | 60 |
| Meningitis aseptic | 4 | 20 | 110 |
| Myelitis transverse | 2 | 8 | 45 |
| Meningoencephalitis | 2 | 7 | 35 |
| Notes: Counts represent distinct case reports for each subtype. | | | |
