# Supplementary material for: Heterogeneity of immune checkpoint inhibitor-related inflammatory central nervous system adverse event reporting signals in primary and metastatic brain tumors: a pharmacovigilance study with single-cell and spatial transcriptomic contextualization
Source: Front Immunol. 2026 Jul 8;17:1866830. doi: 10.3389/fimmu.2026.1866830 (PMC13388250; doi:10.3389/fimmu.2026.1866830)
Supplement: Supplementary file 6 [file Table6.docx]

| **Table S6. Seriousness outcomes by subtype.** | | | | |
| --- | --- | --- | --- | --- |
| irAE Subtype | Severity | Primary CNS (n/N) | Brain Metastases (n/N) | Non-CNS (n/N) |
| Encephalitis | Death | 2/10 (20.0%) | 12/60 (20.0%) | 22/220 (10.0%) |
| Encephalitis | Life-Threatening | 3/10 (30.0%) | 18/60 (30.0%) | 45/220 (20.4%) |
| Meningitis | Death | 0/5 (0.0%) | 2/25 (8.0%) | 5/140 (3.5%) |
| Meningitis | Life-Threatening | 1/5 (20.0%) | 4/25 (16.0%) | 12/140 (8.5%) |
| Myelitis | Death | 1/3 (33.3%) | 3/10 (30.0%) | 8/50 (16.0%) |
| Myelitis | Life-Threatening | 0/3 (0.0%) | 2/10 (20.0%) | 5/50 (10.0%) |
| Notes: Stratification of severe clinical outcomes to assess differential subtype toxicity. | | | | |
