# Supplementary material for: Heterogeneity of immune checkpoint inhibitor-related inflammatory central nervous system adverse event reporting signals in primary and metastatic brain tumors: a pharmacovigilance study with single-cell and spatial transcriptomic contextualization
Source: Front Immunol. 2026 Jul 8;17:1866830. doi: 10.3389/fimmu.2026.1866830 (PMC13388250; doi:10.3389/fimmu.2026.1866830)
Supplement: Supplementary file 7 [file Table7.docx]

**Table S7. Gene composition of the primary and alternative inflammatory/stress modules.**

| **Module** | **Functional category** | **Genes** | **No. of genes** |
| --- | --- | --- | --- |
| Primary strict inflammatory module | IFN / interferon-stimulated signaling | STAT1, IRF1, IFIT1, IFIT3 | 4 |
| Primary strict inflammatory module | Antigen processing and presentation | B2M, TAP1, CD74, HLA-DRA, HLA-DRB1 | 5 |
| Primary strict inflammatory module | Inflammatory chemotaxis | CXCL9, CXCL10, CXCL11, CCL2, CCL5 | 5 |
| Primary strict inflammatory module | Inflammatory activation / adhesion | TNF, TNFAIP3, NFKBIA, ICAM1, VCAM1, IL1B | 6 |
| Primary broad stress module | Hypoxia / angiogenic / metabolic stress | HIF1A, VEGFA, CA9, LDHA, SLC2A1, ADM, SERPINE1 | 7 |
| Primary broad stress module | Immediate early / stress-response transcription | JUN, FOS, ATF3 | 3 |
| Primary broad stress module | Heat-shock / unfolded-protein / injury-associated stress | DDIT3, HSPB1, HSPA1A, CXCL8, PTGS2 | 5 |
| Alternative inflammatory module | Broader IFN / chemotaxis / antigen-presentation program | IFNG, STAT1, STAT2, IRF1, IRF7, ISG15, IFIT1, IFIT2, IFIT3, OAS1, OAS2, MX1, GBP1, GBP2, CXCL9, CXCL10, CXCL11, CCL2, CCL5, B2M, TAP1, TAP2, CD74, HLA-A, HLA-B, HLA-C, HLA-DRA, HLA-DRB1, HLA-DPA1, HLA-DPB1, TNF, IL1B, NFKBIA, TNFAIP3, ICAM1, VCAM1 | 36 |
| Alternative stress module | Broader hypoxia / glycolysis / UPR / immediate early stress program | HIF1A, VEGFA, CA9, BNIP3, EGLN3, ADM, SERPINE1, SLC2A1, LDHA, ENO1, PGK1, HK2, PDK1, DDIT3, ATF3, XBP1, HSPA1A, HSPA1B, HSP90AA1, HSP90AB1, HSPB1, DNAJB1, JUN, FOS, EGR1, DUSP1, CXCL8, PTGS2 | 28 |

Notes: The primary strict inflammatory and primary broad stress modules were used for the main module scoring. The alternative inflammatory and alternative stress modules were used for sensitivity analyses. These modules are compact, literature-informed transcriptional programs and are not intended to serve as irAE-specific diagnostic signatures. IFN, interferon; UPR, unfolded protein response; irAE, immune-related adverse event.
