# Supplementary material for: Heterogeneity of immune checkpoint inhibitor-related inflammatory central nervous system adverse event reporting signals in primary and metastatic brain tumors: a pharmacovigilance study with single-cell and spatial transcriptomic contextualization
Source: Front Immunol. 2026 Jul 8;17:1866830. doi: 10.3389/fimmu.2026.1866830 (PMC13388250; doi:10.3389/fimmu.2026.1866830)
Supplement: Supplementary file 8 [file Table8.docx]

**Table S8. Sensitivity analyses using alternative module definitions and residual-based relative inflammatory scoring across single-cell datasets.**

| **Dataset** | **Sensitivity metric** | **Metric definition** | **Top-scoring compartments** | **Spearman rho vs primary strict-minus-broad** | **P value** | **Interpretation** |
| --- | --- | --- | --- | --- | --- | --- |
| GSE131928 Smart-seq2 | Primary residual inflammatory score | Residuals from regressing primary strict inflammatory z-score on primary broad stress z-score | Myeloid, T/NK | 0.79 | <0.001 | Preserved immune-compartment enrichment |
| GSE131928 Smart-seq2 | Alternative minus-stress score | Alternative inflammatory z-score minus alternative stress z-score | T/NK, Myeloid | 0.84 | <0.001 | Preserved immune-compartment enrichment |
| GSE131928 Smart-seq2 | Alternative residual inflammatory score | Residuals from regressing alternative inflammatory z-score on alternative stress z-score | Myeloid, T/NK | 0.53 | <0.001 | Preserved immune-compartment enrichment |
| GSE131928 10x | Primary residual inflammatory score | Residuals from regressing primary strict inflammatory z-score on primary broad stress z-score | T/NK, Myeloid | 0.70 | <0.001 | Preserved immune-compartment enrichment |
| GSE131928 10x | Alternative minus-stress score | Alternative inflammatory z-score minus alternative stress z-score | T/NK, Myeloid | 0.89 | <0.001 | Preserved immune-compartment enrichment |
| GSE131928 10x | Alternative residual inflammatory score | Residuals from regressing alternative inflammatory z-score on alternative stress z-score | Myeloid, T/NK | 0.68 | <0.001 | Preserved immune-compartment enrichment |
| GSE131907 mBrain | Primary residual inflammatory score | Residuals from regressing primary strict inflammatory z-score on primary broad stress z-score | Myeloid, T/NK | 0.77 | <0.001 | Preserved immune-compartment enrichment |
| GSE131907 mBrain | Alternative minus-stress score | Alternative inflammatory z-score minus alternative stress z-score | T/NK, Myeloid | 0.93 | <0.001 | Preserved immune-compartment enrichment |
| GSE131907 mBrain | Alternative residual inflammatory score | Residuals from regressing alternative inflammatory z-score on alternative stress z-score | Myeloid, T/NK | 0.70 | <0.001 | Preserved immune-compartment enrichment |

Notes: The primary strict-minus-broad score was defined as the within-dataset standardized primary strict inflammatory score minus the within-dataset standardized primary broad stress score. Residual inflammatory scores were calculated by regressing the corresponding inflammatory z-score on the corresponding stress z-score within each dataset and using the residuals as relative inflammation-dominant scores. Top-scoring compartments were defined according to median compartment-level scores. P values correspond to Spearman correlation tests and are reported as P < 0.001 when smaller than 0.001. The sensitivity analyses supported moderate-to-strong concordance with the primary strict-minus-broad metric and preserved immune-compartment enrichment, particularly in myeloid and/or T/NK compartments.
