# Supplementary figures and images for: Heterogeneity of immune checkpoint inhibitor-related inflammatory central nervous system adverse event reporting signals in primary and metastatic brain tumors: a pharmacovigilance study with single-cell and spatial transcriptomic contextualization
Source: Front Immunol. 2026 Jul 8;17:1866830. doi: 10.3389/fimmu.2026.1866830 (PMC13388250; doi:10.3389/fimmu.2026.1866830)

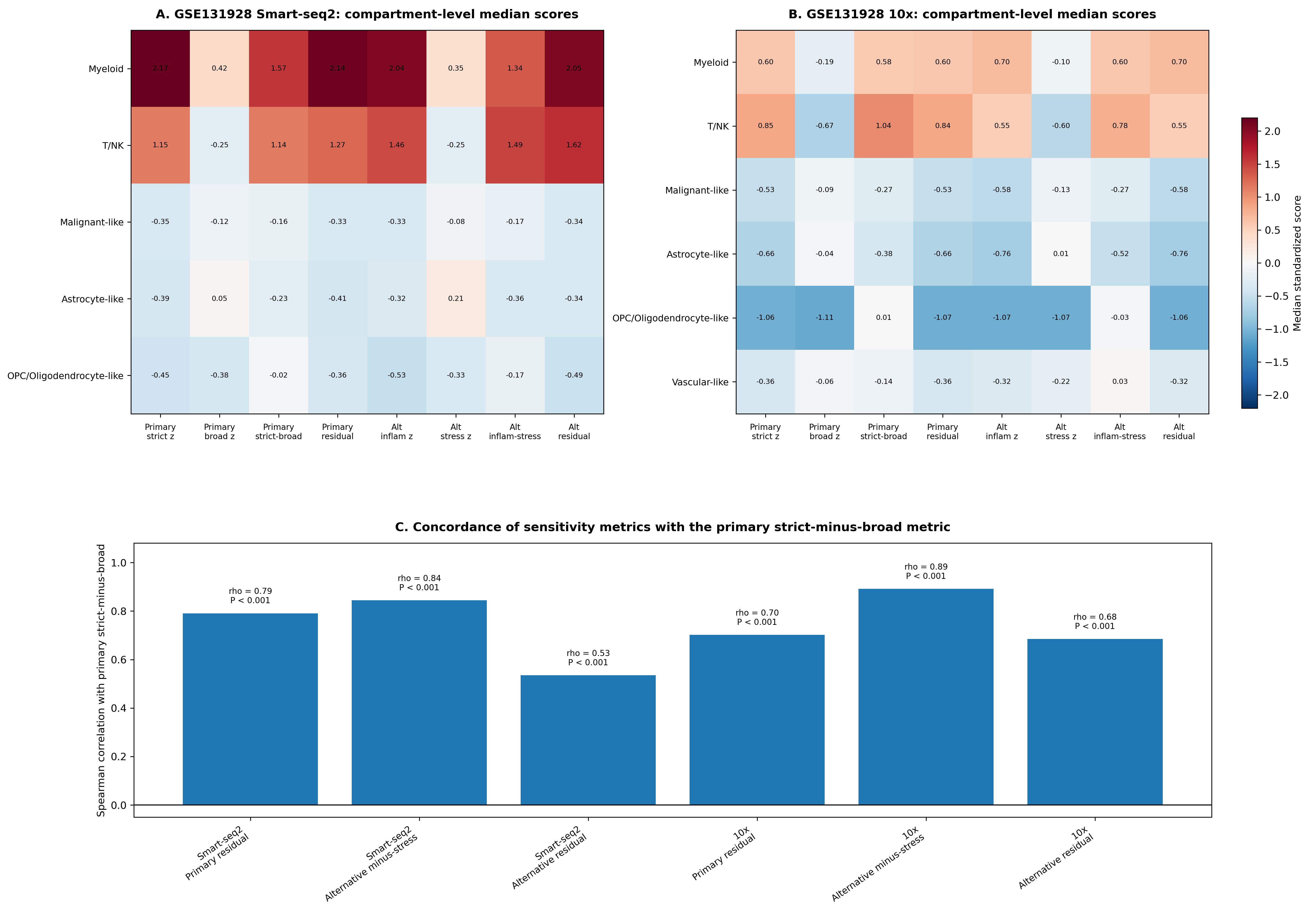

Supplement: Supplementary file 10 [file Image1.tif]

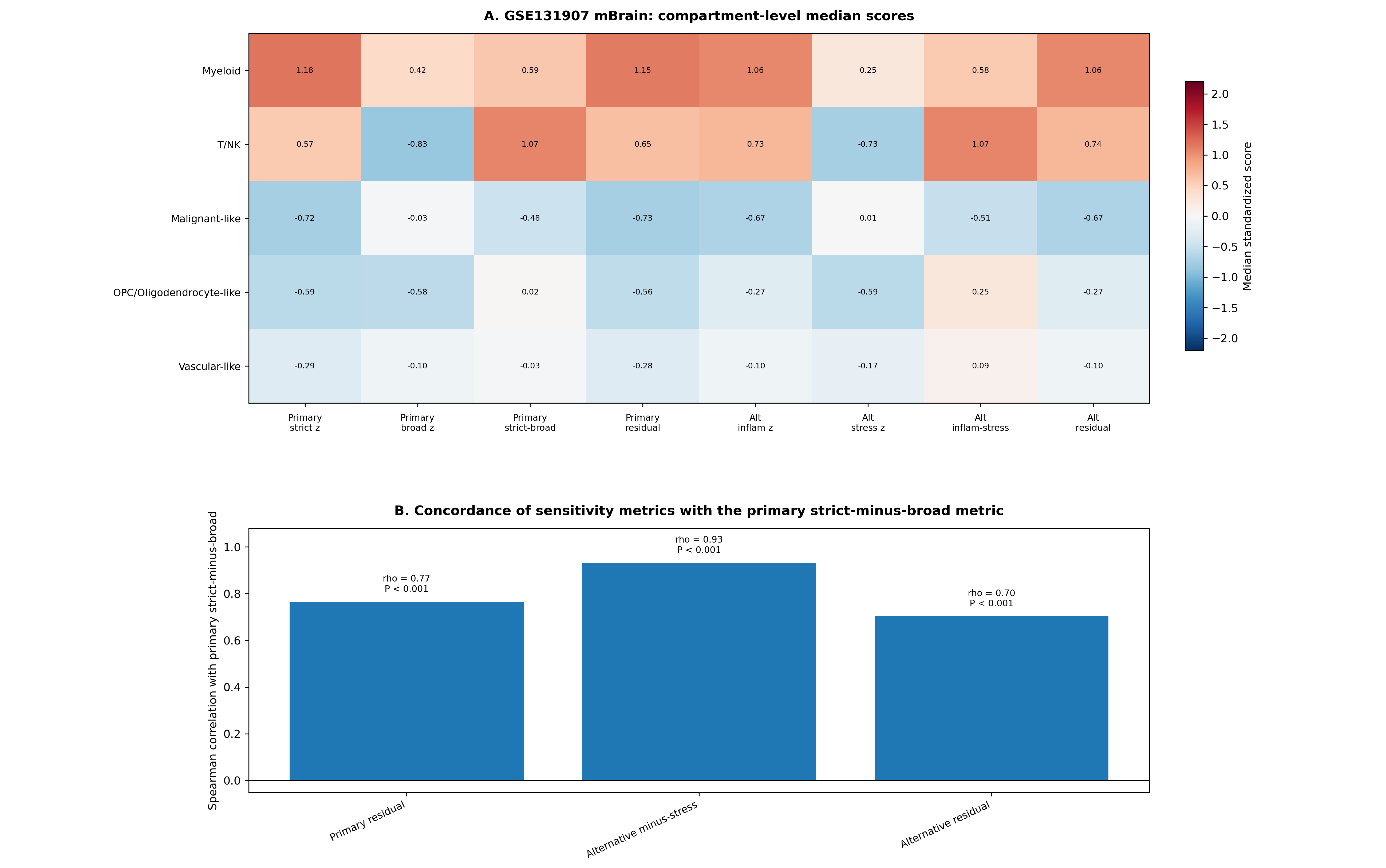

Supplement: Supplementary file 11 [file Image2.tif]

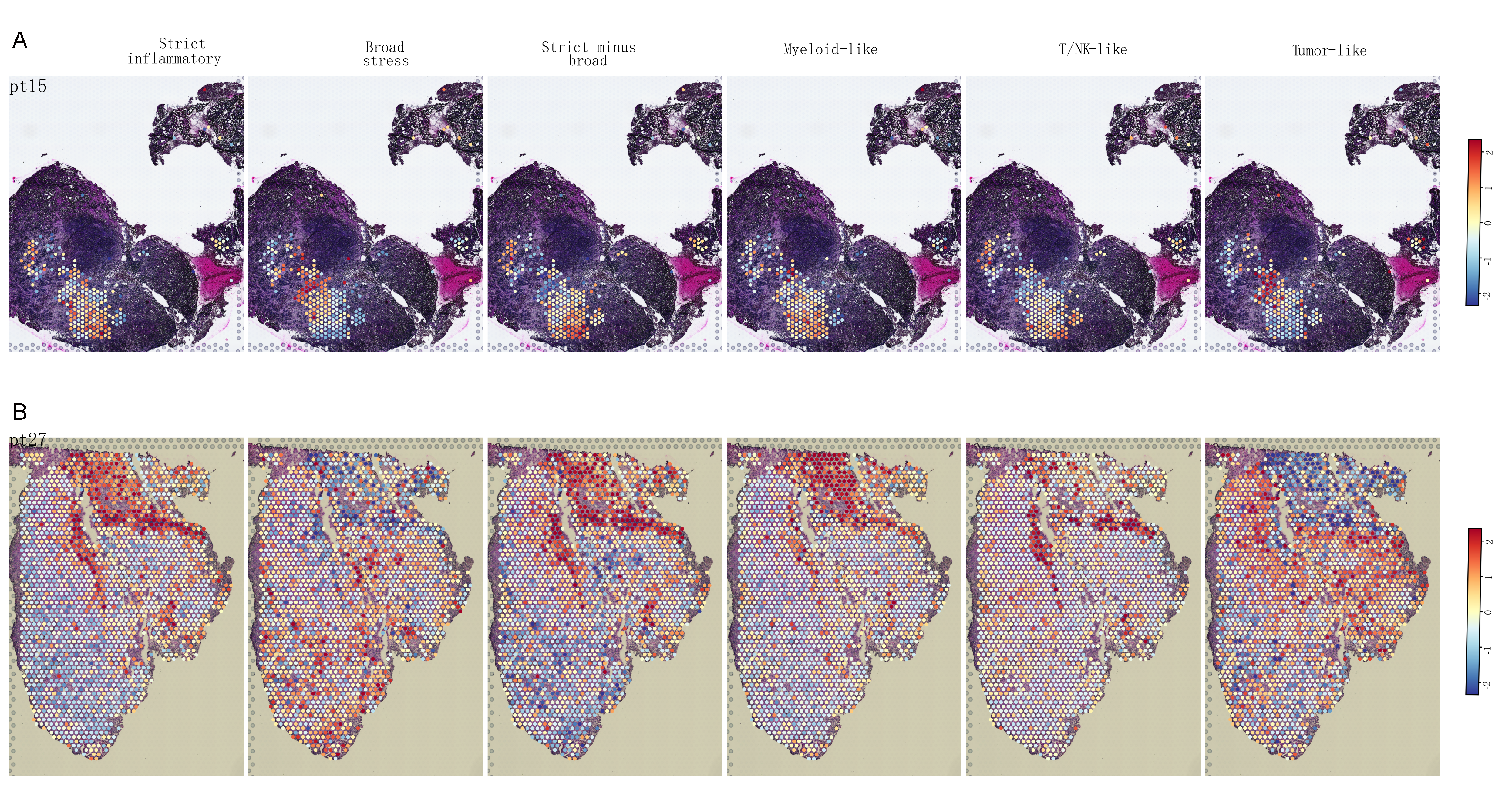

Supplement: Supplementary file 12 [file Image3.tif]

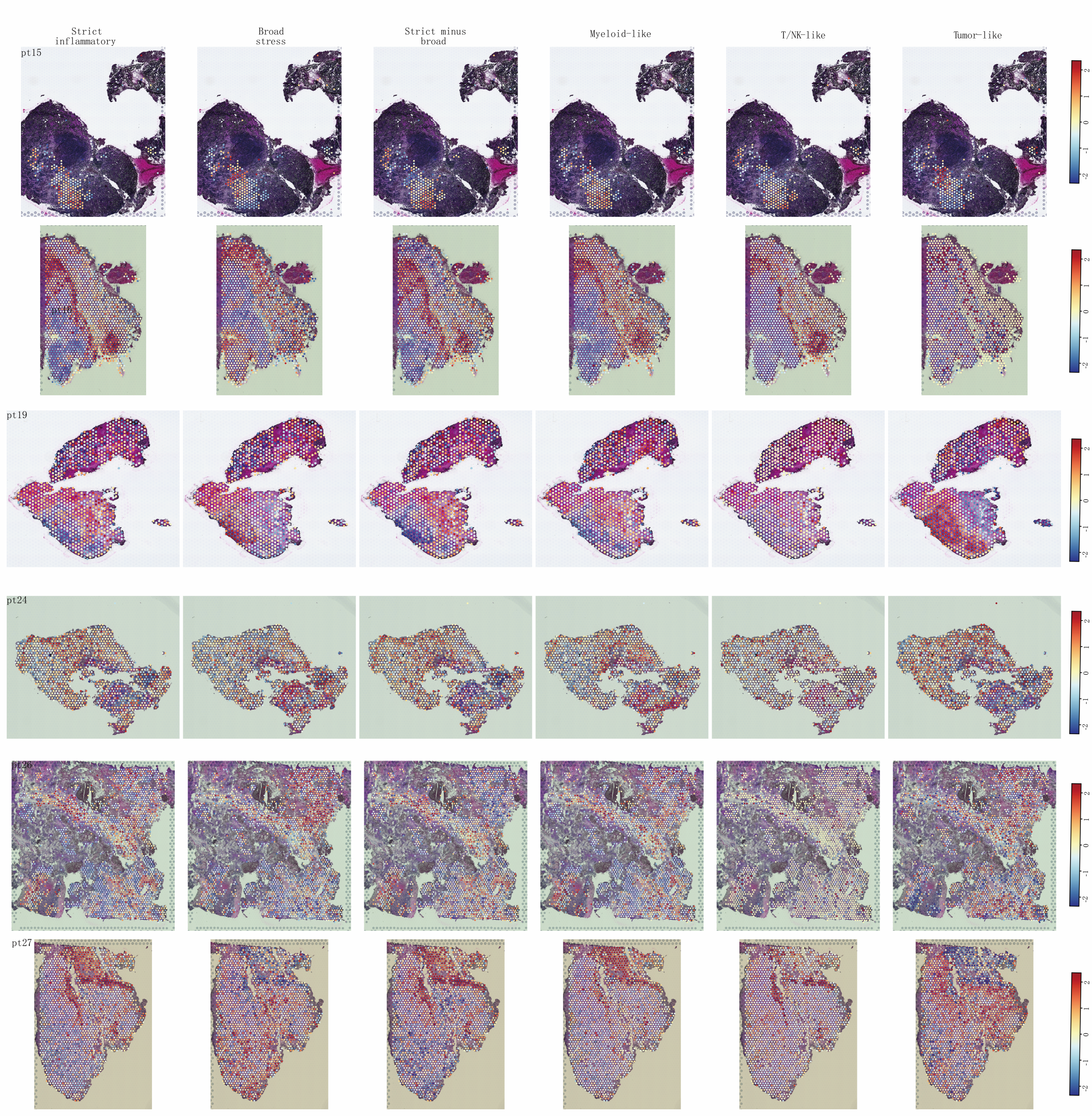

Supplement: Supplementary file 13 [file Image4.tiff]

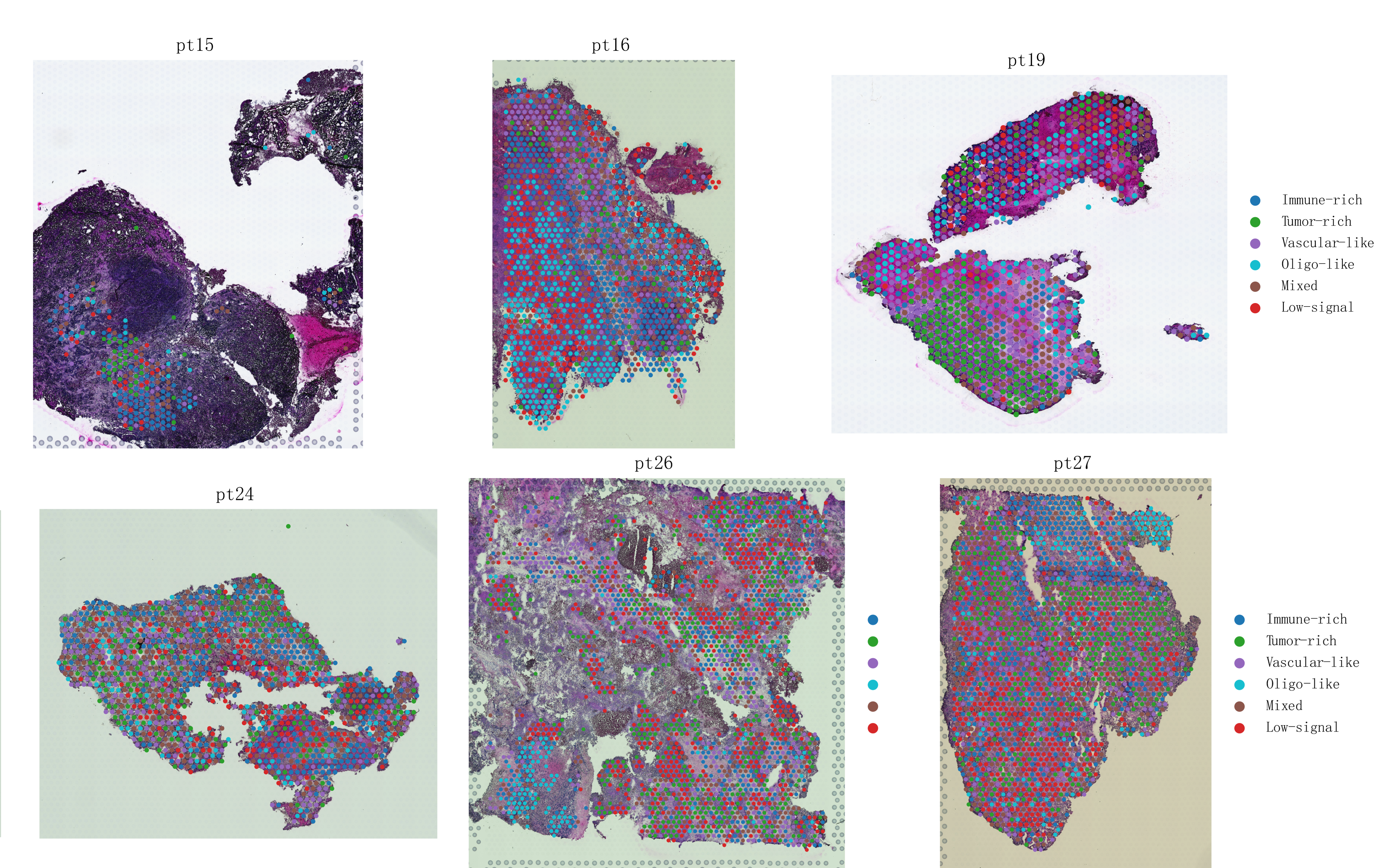

Supplement: Supplementary file 14 [file Image5.tif]
